# A novel multiomics machine learning signature identifies rapid progression in clinically low risk prostate cancer

**DOI:** 10.1038/s41746-026-03254-5

**Published:** 2026-09-14

**Authors:** Alexandra Rafeletou, Faezeh Fathi, Tatjana Kiseļova, Golnaz Taheri, Arian Lundberg

**Affiliations:** 1 Department of Protein Science, KTH Royal Institute of Technology, Stockholm, Sweden; 2 Science for Life Laboratory (SciLifeLab), KTH Royal Institute of Technology, Stockholm, Sweden; 3 School of Electrical Engineering and Computer Science, KTH Royal Institute of Technology, Stockholm, Sweden; 4 School of Electrical Engineering and Computer Science, Digital Futures, KTH Royal Institute of Technology, Stockholm, Sweden; 5 School of Engineering Sciences in Chemistry, Biotechnology and Health, Digital Futures, KTH Royal Institute of Technology, Stockholm, Sweden

**Keywords:** Biomarkers, Cancer, Computational biology and bioinformatics, Oncology

## Abstract

Risk stratification in primary prostate cancer remains heavily reliant on clinicopathological criteria that frequently miss the heterogeneity underlying early aggressive disease. We present a novel machine learning non-linear prognostic framework encoding somatic copy-number alterations and biological information associated with gene products, along with an integrative multi-omics approach including epigenomics and transcriptomics into a patient-specific biological network. Applied to the TCGA-PRAD (*n* = 498), our weighted graph-based feature selection and LASSO-Cox model identified *ZNF268* as a master regulator gene, in which the hypermethylation of its promoter region is linked to a distinct oncogenic transition exclusive to Low/Intermediate-risk disease. Post-hoc analysis of Low-*ZNF268* tumors showed a distinct somatic landscape enriched for driver mutations and predicted sensitivity to MAPK, ATR, and PI3K/mTOR inhibitors, providing potential therapeutic vulnerabilities alongside the prognostic signal. Topological network analysis further revealed that *ZNF268* loss impacts a co-expression rewiring gene network, quantified as a Rewiring Score: associated with Progression-Free Survival in the TCGA-PRAD (HR: 2.79, 95% CI: 1.36–5.71, *p* = 0.0049) and Biochemical Recurrence in two external cohorts. By capturing tumors at an active molecular transition state preceding systemic progression, this framework offers a prognostic tool to identify biologically aggressive prostate cancer disease within patients currently undertreated by standard risk criteria.

## Introduction

Prostate cancer (PCa) remains the second most frequently diagnosed malignancy among men globally^1,2^. While primary disease is often manageable, a distinct subset of patients progresses to lethal metastatic stages^3^. Distinguishing these high-risk patients from those with indolent disease at the time of diagnosis is a critical clinical challenge. Risk stratification tools, such as the National Comprehensive Cancer Network (NCCN) guidelines, rely heavily on static clinicopathological features including biopsy Gleason scores, clinical Tumor-stage, and preoperative serum prostate-specific antigen (PSA) levels. While useful for broad categorizations, these traditional methods frequently fail to account for the substantial intra-tumor molecular heterogeneity within clinically favorable groups^4,5^.

Despite the fact that multi-omics profiling offers a promising solution, traditional analytic approaches that interpret genomic and transcriptomic layers sequentially often miss the synergistic interactions driving pathogenesis. To address this, we performed a multi-omics investigation and developed a machine learning framework to interrogate primary PCa within the context of biological networks. Advanced machine learning architectures offer a decisive advantage over conventional linear statistics by efficiently managing high-dimensional multi-omics datasets, resolving complex non-linear feature interactions, and modeling topological network dependencies without overfitting. Recent implementations have demonstrated how machine learning pipelines can integrate disparate transcriptomic datasets to discover robust shared signatures across distinct patient cohorts^6^, systematically map tumor microenvironment and metabolic heterogeneity^7^, and expose critical biological transition trajectories, such as cell death or network rewiring states, to reveal targetable therapeutic vulnerabilities^8^. Unlike standard linear analyses, this approach integrates heterogeneous multi-omics data to reveal latent regulatory signals and immune-metabolic shifts that sequential omics approaches overlook.

In this study, our novel framework identifies the loss of *ZNF268* as a novel driver of aggressive disease. We demonstrate the prognostic utility of *ZNF268* across large independent cohorts and reveal that epigenetic silencing and network rewiring of *ZNF268* dictate aggressive trajectories at very early-stage PCa. Crucially, we show that the protective effect of this network is highly biologically dependent, providing significant risk stratification within patients classified as Low to Intermediate risk by NCCN guidelines. By identifying early molecular divergence toward aggressive disease within these seemingly safe clinical subgroups, our framework offers a tool to guide early and targeted intervention.

## Results

### NCCN prostate cancer risk groups are associated with distinct genomic profiles and survival outcomes

We stratified patients in the TCGA cohort (*n* = 498) and the MSKCC cohort (*n* = 181) into risk groups according to NCCN guidelines: Low, Intermediate-favorable, Intermediate-unfavorable, High, and Very High risk^9^ (Fig. S1a). However, Kaplan-Meier survival analyses in both cohorts showed no significant differences between Low-risk and Intermediate-risk (favorable or unfavorable) patients, nor between High- and Very High-risk patients (Figures S1b, S1c, S1d). Subsequently, we merged Low and Intermediate groups into a single category (Low/Intermediate) and High and Very High-risk groups into a second category (High/Very High-risk) (Fig. S2a, S2b).

**Fig. 1:**
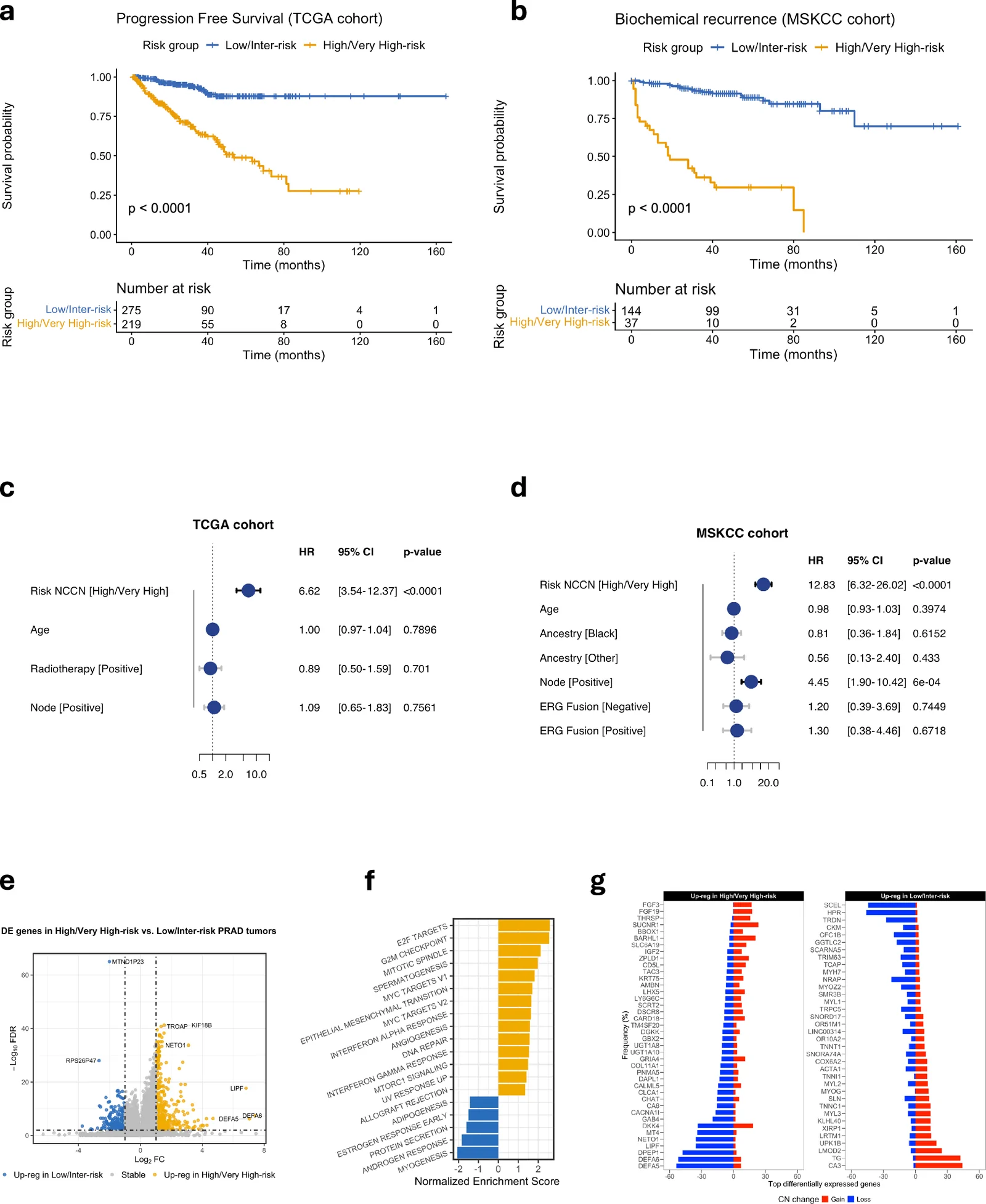
NCCN risk groups are associated with distinct genomic profiles and clinical outcomes. **a**, **b** Kaplan-Meier survival analysis demonstrating significantly worse Progression-Free Survival (PFS) in the TCGA-PRAD and Biochemical Recurrence (BCR) in the MSKCC cohort for High/Very High-risk patients. **c**, **d** Forest plots multivariable Cox proportional hazards analysis confirming the NCCN High/Very High-risk group as an independent predictor of poor prognosis in both the TCGA (adjusted for age, lymph node metastasis, radiotherapy) and MSKCC cohorts (adjusted for age, ancestry, lymph node metastasis, ERG fusion). **e**, **f** Differentially Expressed Genes and Gene Set Enrichment Analysis (GSEA) of Hallmark pathways comparing High/Very High-risk to Low/Intermediate-risk tumors. **g** Integration of aggregate GISTIC copy number profiles with differential gene expression, demonstrating that upregulation in the High/Very High-risk group is predominantly associated with copy number deletions, whereas upregulation in the Low/Intermediate-risk group is driven by amplifications.

This classification showed a strong association with clinical outcomes. In the TCGA-PRAD, High/Very High-risk patients had significantly worse Progression-Free Survival (PFS) compared to the Low/Intermediate group (log-rank *p* < 0.0001; Fig. 1a). Consistently, in the MSKCC cohort, High/Very High-risk patients showed significantly higher rates of Biochemical Recurrence (BCR) (log-rank *p* < 0.0001; Fig. 1b).

Multivariable Cox proportional hazards regression analysis confirmed that the NCCN risk group was an independent predictor of poor prognosis after adjustment for clinicopathological information available in the cohorts. In the TCGA-PRAD, the High/Very High-risk group remained significantly associated with worse PFS after adjusting for age, lymph node metastasis, and radiotherapy (HR: 6.62, 95% CI: 3.54–12.37, *p* < 0.0001; Fig. 1c). Similarly, in the MSKCC-cohort, the high-risk group maintained strong prognostic value (HR: 12.83, 95% CI: 6.32–26.02, *p* < 0.0001; Fig. 1d) following adjustment for age, ancestry background, lymph node metastasis, and ERG fusion status.

To identify the molecular mechanisms distinguishing these risk groups, we performed Gene Set Enrichment Analysis (GSEA). High/Very High-risk patients exhibited significant enrichment in pathways associated with cell proliferation and inflammatory signaling, including the Hallmarks of E2F targets, G2M checkpoint, mitotic spindle, DNA repair, and Interferon-α/γ responses. Conversely, showing reduced activity in the androgen response pathway (FDR < 0.05; Fig. 1e, 1f, SData 1 and 2).

To test the robustness of these biological insights, we expanded our GSEA analysis beyond Hallmark gene sets to include Gene Ontology (GO), KEGG, Reactome, and WikiPathways databases^10–12^. Consistent with the Hallmark pathways analysis, the High/Very High-risk group displayed broad and strong enrichment (NES > 2.0) for processes driving cell cycle progression and genomic instability. Key enriched terms included “cell cycle”, “mitotic spindle,” and “retinoblastoma gene in cancer,” confirming a phenotype of uncontrolled proliferation. In contrast, the Low/Intermediate risk group was characterized by the enrichment of structural and myogenic pathways, such as “sarcomere organization,” and “myofibril assembly.” This distinct enrichment profile suggests that Low/Intermediate risk tumors maintain a more differentiated phenotype or retain a higher proportion of normal stromal architecture compared to their high-risk counterparts (Fig. S3a, SData 3).

To determine the genomic drivers of these transcriptional changes, we integrated matched GISTIC copy number profiles with differential expression data. We assessed whether the top upregulated genes in each risk group were associated with the regional somatic copy number alterations (amplifications or deletions). Notably, while a subset of genes upregulated in the High/Very High-risk group exhibited copy number amplification, the majority of highly expressed genes were associated with copy number loss. Conversely, this pattern contrasted with the Low/Intermediate risk group, where upregulation was predominantly driven by copy number gains (Fig. 1g).

Furthermore, to validate the relationship between somatic copy number alterations and gene expression at the individual sample level, we visualized the copy number status of the top differentially expressed genes across individual samples. Consistent with the aggregate GISTIC analysis at the individual sample level, genes significantly upregulated in the High/Very High-risk group frequently exhibited widespread copy number losses (shallow or deep deletions) within high-risk patients, rather than the expected amplifications. Conversely, genes upregulated in the Low/Intermediate risk group were predominantly associated with copy number gains and amplifications (Fig. S3b). This persistent pattern suggests that the aggressive high-risk transcriptional program is driven by alternative regulatory mechanisms independent of direct genomic amplification or deletion.

### Machine learning identifies *ZNF268* as a novel independent prognostic biomarker in low-risk prostate cancer

While the broad transcriptomic and genomic profiles confirm the biological divergence between NCCN risk strata, standard sequential analyses fail to capture the non-linear, patient-specific regulatory networks driving early disease progression. Therefore, we employed our weighted graph-based machine learning framework to identify latent prognostic vulnerabilities hidden within these clinical classifications. We selected the top 100 ranked protein-coding genes within Low/Intermediate and High/Very High-risk tumors (SData 4).

**Fig. 2:**
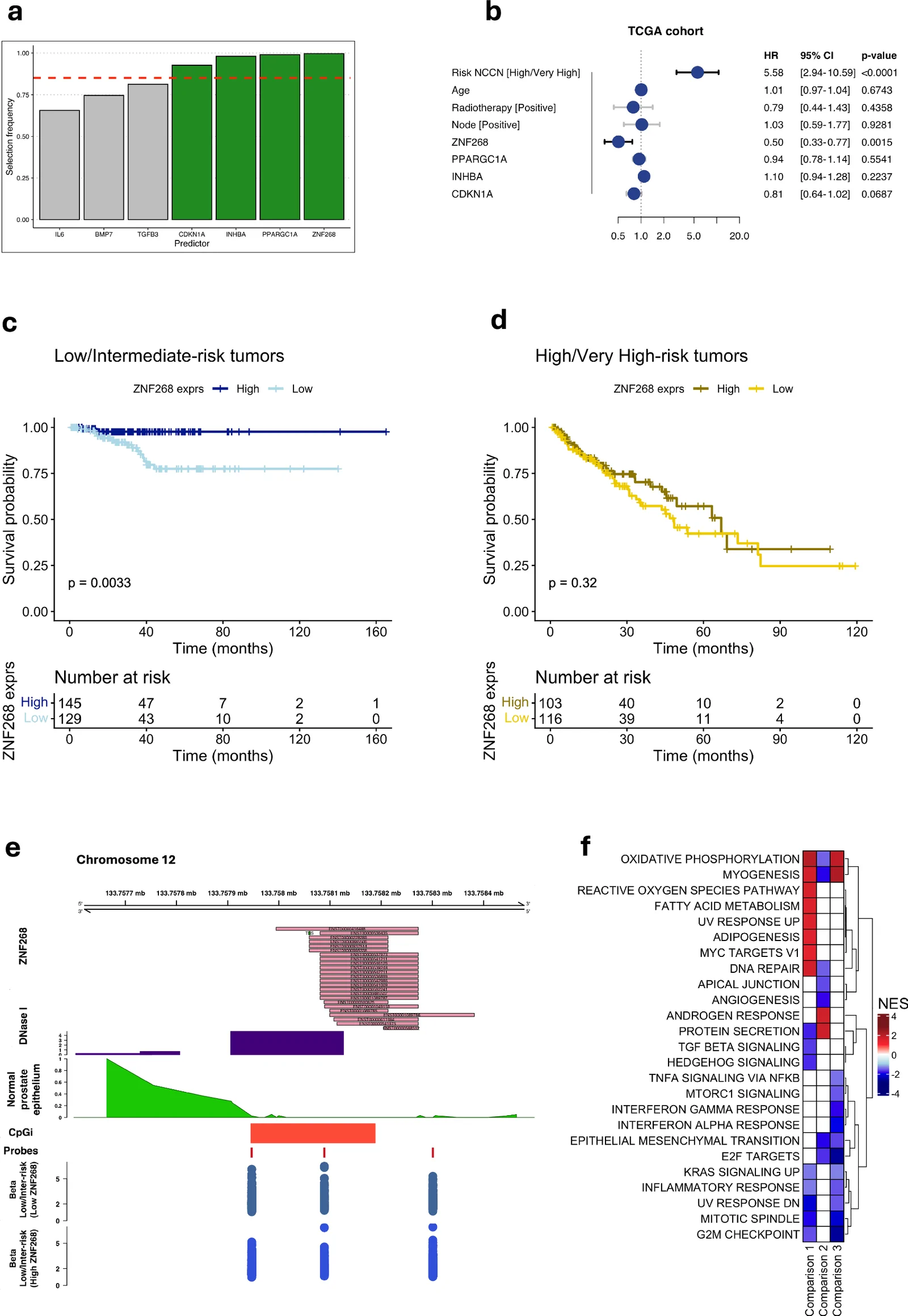
Integrative machine learning identifies ZNF268 epigenetic silencing as a driver of metabolic hyperactivation. **a** Feature selection via a LASSO Cox regression proportional hazards model coupled with bootstrap stability identified a 4-gene prognostic signature. **b** Multivariable Cox regression model evaluating the 4-gene signature alongside standard clinical covariates, identifying *ZNF268* as a robust, independent protective factor. Targeted interaction model (*ZNF268* × NCCN Risk) demonstrating context-dependent prognostic utility. Kaplan-Meier curves show *ZNF268* expression can significantly stratify patients for PFS in the Low/Intermediate-risk group (**c**) but shows no significant stratification in the High/Very High-risk cohort (**d**). **e** Genomic region analysis of normal prostate epithelium displaying an unmethylated, DNase I hypersensitive open chromatin hub at the *ZNF268* regulatory locus. **f** GSEA pathway mapping across clinical trajectories, revealing that early-stage *ZNF268* loss is linked to a phenotypic inversion from structural/immune signaling toward metabolic hyperactivation (Comparison 1: Low/Inter-risk (Low *ZNF268*) vs. Low/Inter-risk (High *ZNF268*), Comparison 2: Low/Inter-risk (High *ZNF268*) vs. High/very High risk tumors, Comparison 3: Low/Inter-risk (Low *ZNF268*) vs. High/very High risk tumors).

To systematically identify the most prognostic hits among the candidate genes driving disease progression, we employed a LASSO Cox proportional hazards regression model (alpha = 1) and a bootstrap stability selection process with 500 iterations, which resulted in the selection of four genes that are associated with PFS: *ZNF268*, *PPARGC1A*, *INHBA*, and *CDKN1A* (Fig. 2a). To validate the clinical relevance of these genes, we first performed univariable Kaplan-Meier survival analysis (Fig. S4), confirming that expression levels of these genes significantly stratify patients by PFS. We then incorporated their expression into a multivariable Cox proportional hazards regression model alongside clinical covariates, including NCCN risk group, patient age, lymph node and radiotherapy status. As expected, the High/Very High NCCN risk classification remained the strongest independent predictor of worse PFS (HR: 5.58, 95% CI: 2.94–10.59, *p* < 0.0001). Notably, within the 4-gene signature, *ZNF268* emerged as the only gene in PCa where its higher expression was strongly and independently associated with improved PFS in both univariable analysis (*p* = 0.0002) and multivariable models (HR: 0.50, 95% CI: 0.33–0.77, *p* = 0.0015) (Fig. 2b).

To evaluate the validity of *ZNF268* as an independent prognostic biomarker, we analyzed an external PCa cohort (Long et al. Cancer Research 2014)^13^. In a multivariable Cox proportional hazards regression model adjusting for key clinical covariates, including Age, Pre-operative PSA, Gleason Score, and Pathological Stage, *ZNF268* expression served as an independent protective factor associated with Biochemical Recurrence-Free survival (HR = 0.60, 95% CI: 0.37–0.97, *p* = 0.0371, Fig. S5).

Having established *ZNF268* as a novel independent protective factor in these cohorts, we next evaluated its expression across clinical disease states. *ZNF268* gene expression level decreased significantly with disease progression, showing a stepwise reduction from normal prostate tissue to Low/Intermediate-risk tumors, and declining further in High/Very High-risk tumors (Fig. S6a).

Given the fundamental biological divergence across NCCN risk strata, we hypothesized that the prognostic utility of *ZNF268* might be modulated by the underlying disease state. To test this, we constructed a targeted interaction model [1]:

1 $${ZNF}268\,{expression}\,\times \,{NCCN}\,{Risk}\,+\,{Available}\,{Clinical}\,{variables}$$

This analysis shows significant interaction (p _interaction_ = 0.027, Fig. S7), indicating that the protective effect of *ZNF268* is biologically dependent. To visualize this dependency, we stratified the patients of TCGA-PRAD in each risk group by the median gene expression level of *ZNF268* over the whole cohort. Subgroup survival analyses confirmed the interaction model showing that within the Low/Intermediate risk group, patients with high *ZNF268* expression exhibited significantly longer PFS compared to those with low expression (*p* = 0.0033, Fig. 2c). Conversely, this stratification showed no significant survival difference within the High/Very High-risk risk group (*p* = 0.32, Fig. 2d).

### Epigenetic dysregulation of *ZNF268* is linked to metabolic hyperactivation

We next investigated the genomic and epigenetic mechanisms underlying this context-dependent dysregulation of *ZNF268*. While the somatic copy number alterations differed significantly between Low/Intermediate-risk and High/Very High-risk groups, these differences did not explain the variation in *ZNF268* expression within Low/Intermediate-risk groups (Fig. S6b copy number difference *p* > 0.05; Fig. S6c correlation between the *ZNF268* CNA and mRNA expression *p* > 0.05).

We therefore hypothesized that epigenetic modifications drive this localized transcriptomic heterogeneity. Analysis of individual CpG probes mapping *ZNF268* gene location revealed that methylation levels at three specific loci (cg02461665, cg24464813, and cg21808250) localized to the *ZNF268* promoter region were significantly elevated in tumors with low *ZNF268* expression in Low/Intermediate-risk tumors (Fig. S8, SData 5). Notably, methylation at cg24464813 showed a stronger negative correlation with *ZNF268* mRNA expression specifically in the Low/Intermediate risk cohort compared to the other loci (Fig. S8b, R = -0.34, *p* < 0.0001), indicating an inverse association between this promoter-region methylation and *ZNF268* expression control.

To understand this, we examined the genomic region surrounding the nearest CpG island in normal prostate epithelium^14^. This baseline analysis revealed a complete absence of methylation, accompanied by an enrichment of DNase I hypersensitive sites^15^, suggesting this open chromatin region acts as a critical enhancer or regulatory element for *ZNF268* (Fig. 2e).

Furthermore, to investigate the biological impacts of *ZNF268* silencing, we performed differential expression and GSEA analysis across clinical subgroups (Fig. 2f, SData 6). Within the early-stage Low/Intermediate-risk cohort, direct comparison of Low *ZNF268* versus High *ZNF268* tumors (Fig. 2f, Comparison 1) suggested significant transcriptional reprogramming. Tumors retaining high *ZNF268* expression maintained baseline immune surveillance and structural signaling, including TGF-β signaling, KRAS signaling, Inflammatory Response, and G2M checkpoints. Conversely, the epigenetic loss of *ZNF268* was associated with a strong phenotypic inversion toward metabolic hyperactivation. Consistent with known metabolic drivers of aggressive PCa, Low/Intermediate-risk Low *ZNF268* early-stage tumors had an upregulation of Oxidative Phosphorylation, Fatty Acid Metabolism, MYC targets, and DNA Repair pathways.

We next mapped the molecular trajectories of these subgroups as they progress toward the advanced High/Very High-risk state. Transitioning from the protective High *ZNF268* state to a High/Very High-risk state (Fig. 2f, Comparison 2) showed a distinct pathway inversion, defined by the suppression of Epithelial Mesenchymal Transition (EMT), E2F targets, Oxidative Phosphorylation, and Angiogenesis, alongside an upregulation in Androgen Response and Protein Secretion. In contrast, pathway divergence took a different form when comparing early-stage Low *ZNF268* tumors directly to High/Very High-risk tumors (Fig. 2f, Comparison 3). This trajectory was characterized by further increases in Oxidative Phosphorylation and Myogenesis, coupled with pronounced down-regulation of immune and cell cycle pathways, including Interferon α/γ responses, TNF-α signaling via NF-kB, and Mitotic Spindle assembly.

Together, these data suggest that *ZNF268* silencing in early-stage disease may be driven by the targeted hypermethylation of a normally accessible regulatory element; this event could imply a critical stage, regulating the metabolic and immunological shift from indolent to aggressive PCa.

### Distinct mutational landscapes and driver genes are dictated by *ZNF268* status

**Fig. 3:**
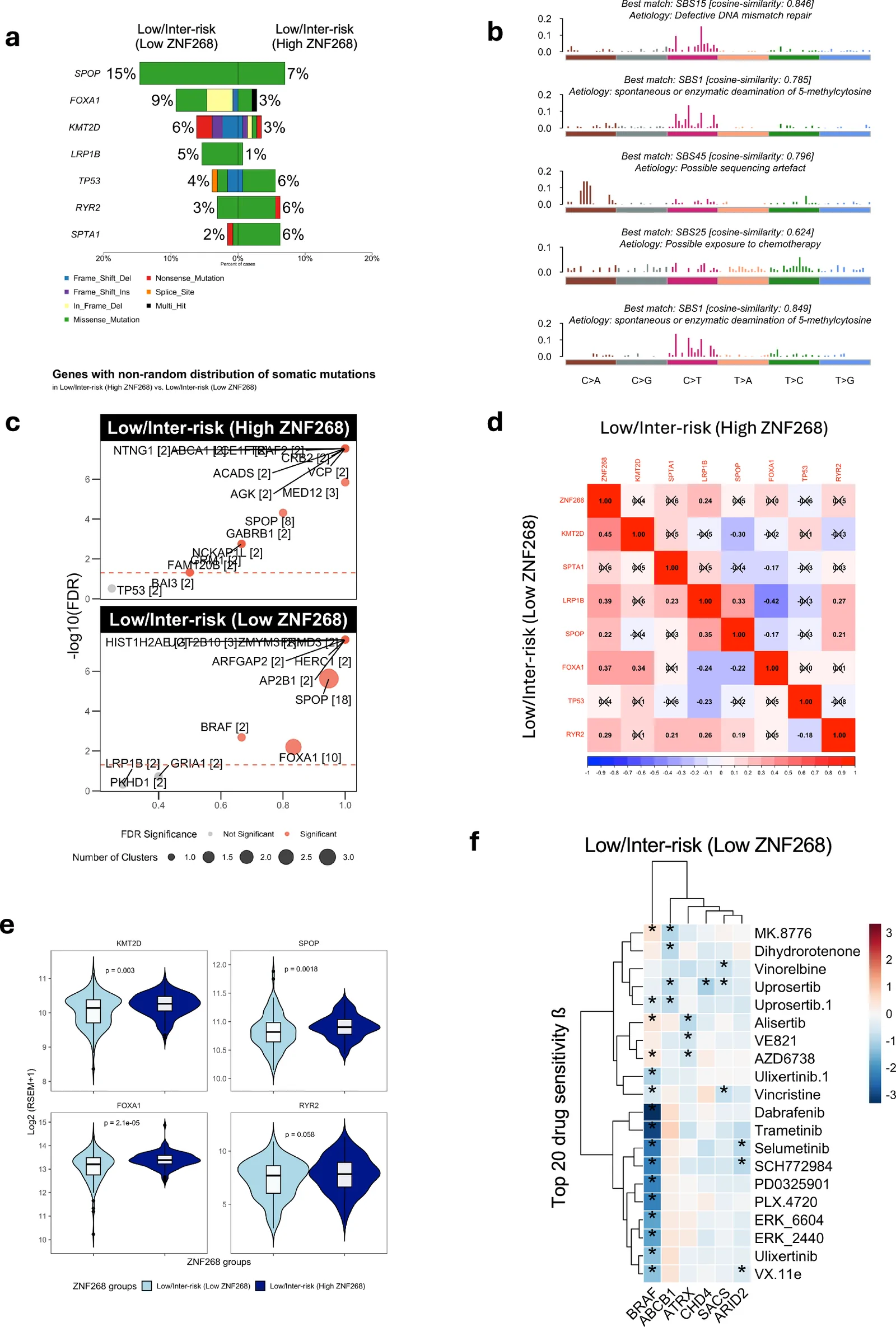
Divergent somatic mutational landscapes and actionable vulnerabilities dictated by *ZNF268* status. **a** Comparative mutational frequency landscape between High *ZNF268* and Low *ZNF268* tumors within the clinical Low/Intermediate-risk tumors, highlighting enrichment of canonical drivers in the Low *ZNF268* subgroup. **b** Mutational signature extraction mapping genomic alterations to distinct etiologies. **c** Non-random mutational clustering analysis distinguishing significant driver events strictly within Low *ZNF268* tumors. **d**, **e** Gene expression correlation matrices and absolute expression levels demonstrating significant transcriptional dysregulation of highly mutated driver genes following *ZNF268* loss. **f** GDSC drug sensitivity profiling. Heatmap-based global vulnerability mapping identifies robust, actionable therapeutic sensitivities unique to the Low *ZNF268* mutational profile, particularly to MAPK pathway inhibitors (e.g., Trametinib), DNA damage response inhibitors (ATR/ATRX targeting), and PI3K/mTOR compounds.

To understand the genomic mechanisms driving the divergent clinical outcomes within the clinical Low/Intermediate-risk patients, we evaluated somatic mutation profiles stratified by *ZNF268* expression. Comparative mutational analyses revealed a markedly distinct somatic landscape in the highly aggressive Low *ZNF268* subgroup. Specifically, these tumors exhibited enriched mutation frequencies in canonical PCa drivers^16^ compared to their High *ZNF268* counterparts, including *SPOP* (15% vs. 7%, Fisher exact *p* < 0.05, OR: 2.26), *FOXA1* (9% vs. 3%, Fisher exact *p* < 0.036, OR: 3.51), and *LRP1B* (5% vs. 1%, Fisher exact *p* < 0.029, OR: 8.03) (Fig. 3a). Mutational signature extraction mapped these genomic alterations to distinct etiology profiles, including defective DNA mismatch repair and spontaneous deamination of 5-methylcytosine (Fig. 3b).

Furthermore, in order to distinguish driver events from passenger mutations, we analyzed the distribution of somatic variants to identify genes with non-random mutational clustering. This analysis revealed distinct mutational landscapes between the risk groups and confirmed that *SPOP* and *FOXA1* act as highly significant, clustered driver mutations strictly within the Low *ZNF268* tumors (FDR < 0.05) (Fig. 3c).

Given the distinct mutational burden, we next investigated the transcriptional interplay between *ZNF268* and these highly mutated driver genes. Gene expression correlation matrices demonstrated different co-expression architectures between the two subgroups, highlighting a context-specific rewiring of transcriptional networks associated with *ZNF268* loss (Fig. 3d). Direct evaluation of the gene expression levels further validated this regulatory divergence. Tumors in the aggressive Low *ZNF268* subgroup demonstrated significant dysregulation of critical driver genes, featuring markedly lower expression of *KMT2D* (*p* = 0.003), *SPOP* (*p* = 0.0018), and *FOXA1* (*p* < 0.0001) compared to the High *ZNF268* baseline (Fig. 3e). This shows that *ZNF268* downregulation is associated not only with somatic mutations in these PCa driver genes but also with their widespread transcriptional dysregulation.

### Drug sensitivity profiling reveals actionable vulnerabilities in Low *ZNF268* Tumors

Standard clinical guidelines often assign conservative, observational strategies to Low/Intermediate-risk patients. However, because our Low *ZNF268* signature identified a hidden subset of biologically aggressive tumors, we sought to identify potential targeted therapeutic options for these high-risk individuals. Using the Genomics of Drug Sensitivity in Cancer (GDSC) database^17^, we mapped the mutational signatures and mutated genes unique to the Low *ZNF268* group against in vitro drug screening betas to predict compound sensitivity.

Clustering of the top 20 sensitivity profiles identified a vulnerability landscape unique to the Low *ZNF268* mutational profile (Fig. 3f). Evaluating the overall vulnerability landscape highlighted strong predicted sensitivities (indicated by highly negative mean beta coefficients) across several critical signaling axes (Fig. S9a). Most notably, Low *ZNF268* tumors showed significant predicted sensitivity to MAPK pathway inhibitors (e.g., Dabrafenib, Trametinib, Selumetinib) driven by BRAF and related mutations, as well as significant vulnerability to DNA damage response inhibitors (e.g., VE821, AZD6738 targeting ATR/ATRX) and PI3K/mTOR targeting compounds (Fig. S9b).

To further test whether the 20 candidate drugs interact within the immediate network neighborhood of *ZNF268*, we performed network proximity analysis on the high-confidence STRING human interactome (combined score > 700) following the methodology introduced by Guney et al.^18^

The analysis revealed that several genes targeted by our prioritized drugs (Fig. S9) are significantly localized near *ZNF268* within the Protein-Protein Interaction (PPI) network. High-ranking candidates, including Dabrafenib, Selumetinib, Alisertib, and Vincristine, exhibited observed distances (*d*_obs_) that were markedly shorter than expected under degree-preserved null models, achieving strong statistical significance (*Z* ≤ −3.0, *p* = 0.002). Conversely, non-significant candidates showed target distances similar to random network expectation (${\mu }_{{null}}$). The complete metrics, including target count, observed distance (*d*_obs_), expected null distance (${\mu }_{{null}}$), Z-score, and empirical *p*-value p-value, for all 20 candidate drugs are detailed in SData 7.

These data suggest that while standard clinical metrics underestimate the risk of Low *ZNF268* tumors, their unique biology harbors distinct, targetable vulnerabilities that could be leveraged for early intervention.

### Dynamic topological reconfiguration of the *ZNF268* co-expression network

**Fig. 4:**
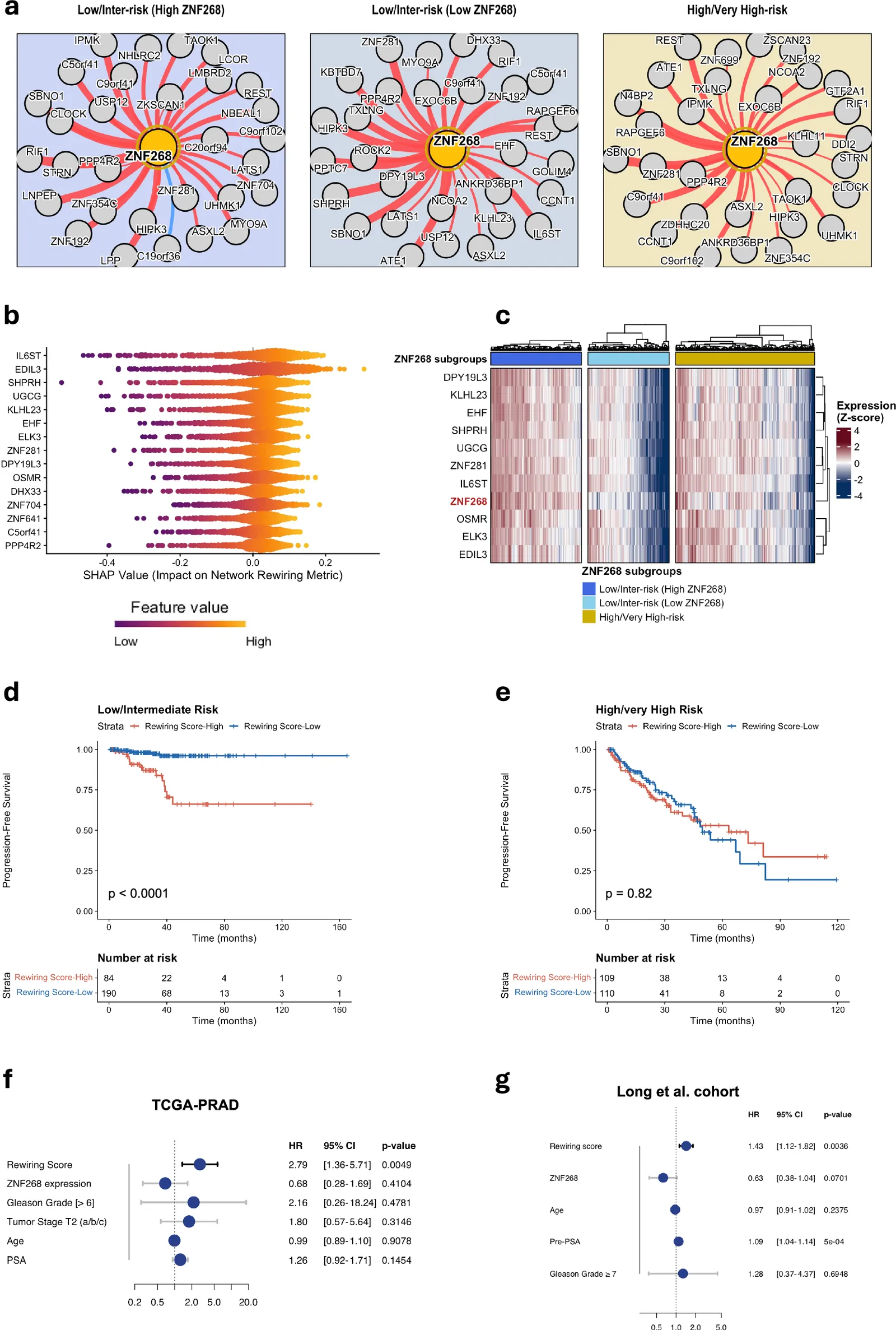
Dynamic topological rewiring of the *ZNF268* network captures an active transitional state. **a** Correlation-based *ZNF268* co-expression networks mapped across three disease states, demonstrating structural turnover and recruitment of distinct gene modules. Positive Pearson’s correlation is shown in red; negative correlation in blue. The width of the line represents the strength of the correlation. **b** Assessing the sample-specific contributions of the genes through a post-hoc machine learning SHAP interpretation. **c** Heatmap visualization of the 15-gene core expression. (Kaplan-Meier analysis of the derived Rewiring Score, effectively stratifying PFS in the Low/Intermediate-risk group (**d**) while showing attenuated stratification in the High/Very High-risk group (**e**). Forest plots representing multivariable Cox regression models including the Rewiring Score in the TCGA-PRAD (**f**) and Long et al. cohort (Cancer Research 2014) (**g**) along with available clinical variables.

To understand how *ZNF268* regulates PCa progression, we evaluated the dynamic topological shifts within its co-expression network by constructing correlation-based networks across three distinct disease states from Low/Intermediate-risk (High *ZNF268*) and Low/Intermediate-risk (Low *ZNF268*), to High/Very High-risk patients. As the disease progresses from the baseline state (Low/Intermediate-risk, High *ZNF268*) into the transitional Low/Intermediate-risk Low *ZNF268* state, and ultimately to the High-risk phenotype, we observe a distinct turnover in the ZNF268-hub architecture. Specific co-expressed genes are recruited into the *ZNF268* module, including transition mediators such as *EHF* and *ZNF281* that have been linked to lineage plasticity, EMT, and PCa progression^19,20^, while previously correlated homeostatic genes are breaking their connection, indicating that *ZNF268*’s regulatory role is highly context-dependent and evolves with disease severity (Fig. 4a).

This structural shift is quantitatively supported by our correlation decay analysis (Fig. S10a) in which the decay trajectory measures the “rigidity” of the gene network, with Low/Intermediate-risk Low *ZNF268* having a slower decay. This result implies that *ZNF268* is holding a highly coordinated, rigid module of genes. Rather than reflecting a homeostatic regulatory state, this extensive co-regulation is indicative of a molecular bottleneck. These findings suggest that the tumor is undergoing transcriptional rewiring in response to evolutionary pressures, resulting in tighter coupling of key pathways that promote progression to a more aggressive disease state.

We hypothesized that the most critical genes driving this network reformation are those that alter their connectivity with *ZNF268*. By calculating the difference in network connectivity between the High and Low *ZNF268* expression tumors within the Low/Intermediate-risk patients, we quantified this network rewiring (Fig. S10b). Here, a strong negative connectivity signifies a functional uncoupling where the genes that were previously synchronized with *ZNF268* in early-stage disease are either discarded from the regulatory module or actively repressed during the transition state. This suggests a dismantling of the homeostatic transcriptional program to allow for tumor evolution. Pathway enrichment analyses on these rewiring genes confirmed that this uncoupling is not random and is highly enriched for cancer hallmarks, including PI3K/AKT/mTOR signaling, DNA repair pathways, and MYC targets (Fig. S10c).

### The rewiring signature captures a unique intermediate phenotype

To identify key drivers of the *ZNF268* regulatory network while minimizing cohort-specific overfitting, we applied a parallelized bootstrapping approach (100 iterations per split) across two independent 50/50 data partitions of the cohort (Fig. S11a). This analysis yielded a highly stable core of 15 rewiring genes, defined by selection frequency of at least 80% across iterations, including *UGCG*, *ZNF281*, *EHF*, and *IL6ST* (Fig. S11). We then performed a post-hoc machine learning SHAP-based prioritization algorithm^21^ within this stable core to derive the final 10-gene Rewiring Score panel used for downstream sample-level scoring and interpretation of network rewiring events associated with rapid disease progression (Fig. 4b, SData 8). The consistent emergence of these specific targets indicates that *ZNF268* rewiring provides the tumor with the signaling and metabolic adaptation required for progression. The expression pattern of identified rewiring shows that this group represents a transient molecular phenotype, an active bridging state capturing tumors in the molecular inflection point of escaping early-stage homeostatic control and uncoupling their native epithelial constraints (Fig. 4c).

### Context-dependent *ZNF268* rewiring network is associated with aggressive trajectories in clinically low-risk prostate cancer tumors

A major challenge in PCa oncology is identifying which seemingly low-risk tumors harbor the potential for rapid systemic progression. Our network analysis established that *ZNF268* may not function as a static driver of disease; rather, its regulatory influence is biologically context-dependent, which alters its transcriptional network between early and advanced disease states. Since our 10-gene signature captures the direct downstream consequence of this *ZNF268* modulation point and representing tumors in a state of active transcriptional plasticity, we hypothesized that its prognostic utility must inherently mirror this context dependency. Specifically, the signature should exert its strongest predictive power in early-stage tumors actively navigating this ZNF268-mediated evolutionary bottleneck, rather than in advanced tumors that have already established a fixed, aggressive phenotype. To test this, we translated the 10-gene core expression into a weighted “Rewiring Score” as a quantitative measure of a tumor’s transcriptomic transition state.

Kaplan-Meier survival analysis supported our biological rationale, with a high Rewiring Score strongly stratifying patients in the Low/Intermediate-risk TCGA-PRAD cohort for PFS and identifying patients whose tumors appeared to be undergoing active disruption of homeostatic regulatory programs (*p* < 0.0001, Fig. 4d). Conversely, as expected, the prognostic value of the transition signature was markedly attenuated in the High/Very High-risk patients (*p* = 0.82, Fig. 4e). This may indicate that advanced tumors have already undergone the transcriptional rewiring captured by the signature, such that this molecular transition no longer provides sufficient variation to discriminate progression risk within an already aggressive disease state.

We then performed multivariate Cox proportional hazards regression analyses within the early-stage, Low/Intermediate risk patients. The Rewiring Score emerged as a robust, independent driver of PFS, maintaining its significance alongside clinicopathological variables such as Gleason grade score, tumor stage, age, PSA levels and *ZNF268* expression (HR: 2.79, 95% CI: 1.36–5.71, *p* = 0.0049, Fig. 4f). This demonstrates that underlying network plasticity linked to *ZNF268* context acts as determinant of progression, independent of standard morphological and clinical grading. Furthermore, we evaluated the performance of the rewiring score over time by calculating the time-dependent Area under the curve (AUC) within the Low/Intermediate-risk patients. This analysis demonstrated stable accuracy across all longitudinal timepoints, spanning 1 to 5 years. The AUC values for these timepoints were 0.77, 0.78, 0.72, and 0.73, respectively (Fig. S12).

To evaluate our network model across the full biological continuum of disease failure, we next evaluated the Rewiring Score in Long et al. and MSKCC cohorts using BCR as the clinical endpoint. While PFS reflects later-stage, macro-level clinical progression and structural tumor growth, BCR serves as an early, highly sensitive molecular indicator of micrometastatic activity and cellular escape long before physical tumors manifest radiographically. Consistent with the TCGA-PRAD, this network signature remained a significant, independent predictor of BCR within Low/Intermediate-risk in both cohorts, even after adjusting for available clinical and molecular covariates (Long et al. HR: 1.43, 95% CI: 1.12–1.82, *p* = 0.0036 and MSKCC HR: 3.08, 95% CI: 1.12–8.46, *p* = 0.0289, cohorts respectively; Fig. 4g, Fig. S13).

Ultimately, by quantifying the biologically context-dependent, transient network rewiring of *ZNF268*, we could identify clinically low-risk PCa patients who are already bound to an aggressive molecular trajectory.

## Discussion

Risk stratification in primary prostate cancer (PCa) remains heavily reliant on clinicopathological parameters, such as NCCN guidelines, which may fail to capture the underlying molecular heterogeneity driving early aggressive disease. Standard linear omics analyses often obscure the dynamic, non-linear regulatory networks that dictate tumor evolution. In this study, we developed a novel integrative machine learning framework to map the molecular trajectory of PCa progression. We identified the biologically context-dependent silencing of *ZNF268* as an independent driver of poor prognosis and discovered that its topological network rewiring serves as a critical evolutionary bottleneck, capturing tumors in an active, targetable “transitioning state” prior to systemic progression.

The identification of *ZNF268* as a robust protective factor is a highly novel finding. While *ZNF268* belongs to the Krüppel-associated box (KRAB) domain-containing zinc finger family, traditionally recognized as transcriptional repressors^22^, recent evidence suggests specific isoforms are vital for amplifying innate immune responses and stabilizing signaling complexes^23^. Our multi-omics analysis provides a timeline for its dysregulation in PCa. We found that *ZNF268* silencing is not merely a late-stage consequence of genomic instability, but an active, early-stage event. In normal prostate epithelium, the *ZNF268* regulatory region exists as an open, accessible chromatin region. We discovered that within the otherwise indolent Low/Intermediate-risk PCa group, targeted hypermethylation of a specific promoter region of *ZNF268* drives its suppression. This epigenetic event links to a phenotypic inversion, prematurely shifting the tumor from a protective, immune-active baseline toward an aggressive, MYC-driven, and metabolically hyperactive state characterized by enhanced oxidative phosphorylation and lipid metabolism.

Importantly, the loss of *ZNF268* was linked to the tumor’s foundational genomic landscape. The highly aggressive Low *ZNF268* subgroup exhibited a distinct mutational architecture, heavily enriched for clustered driver mutations in *SPOP* and *FOXA1*, alongside markers of defective DNA mismatch repair^24^. This localized genomic instability suggests that as tumors acquire specific mutational burdens, the protective *ZNF268* immune-metabolic axis is functionally dismantled. Furthermore, identifying this collapse exposes specific therapeutic vulnerabilities. Our drug sensitivity profiling revealed that these biologically high-risk, Low *ZNF268* tumors harbor distinct actionable susceptibilities, particularly to MAPK pathway inhibitors and DNA damage response targeting compounds (e.g., ATR inhibitors). This provides a rationale for leveraging these distinct mutational profiles for early, targeted interventions in patients who would otherwise be relegated to conservative observation.

Our topological network analysis revealed that the transition from a stable baseline to an advanced phenotype requires a massive structural reorganization of the *ZNF268* co-expression module. The delayed correlation decay observed in the Low *ZNF268* transitional group indicates a period of intense topological rigidity. Rather than reflecting a relaxed network, this tightening is characteristic of a molecular bottleneck that may cause the tumor to actively govern a core transitional program and overcome selective pressures.

Through parallelized bootstrapping, we captured this exact inflection point, isolating a highly stable 10-gene rewiring core (≥80% of the time). The consistent emergence of transition mediators, such as *ZNF281* and *EHF* (master regulators of epithelial-mesenchymal plasticity), *IL6ST* (inflammatory signaling), and *UGCG* (lipid metabolic reprogramming), proves that this rewiring supports the tumor for systemic escape^19,20,25^. Crucially, the expression of these genes highlighted a unique, non-linear bridging state. These results support the idea that progression is not a simple stepwise accumulation of errors but requires cells to pass through a distinct phase of profound transcriptional plasticity to uncouple their native epithelial constraints.

Because this signature is derived directly from the biological inflection point of disease, its predictive power is inherently linked to early-stage tumors actively navigating this evolutionary bottleneck. The score significantly stratified PFS in the Low/Intermediate-risk TCGA-PRAD and proved to be a predictor of BCR in the Long et al. and MSKCC cohorts. As expected biologically, this stratification was attenuated in High/Very High-risk tumors, as these advanced tumors have already bypassed this specific transitional phase and established a fixed aggressive phenotype. This highlights the risk of applying universal biomarkers across all disease stages and underscores the necessity of context-aware prognostic modeling.

A primary methodological strength of our study is the integration of multi-omics and biologically informed machine learning. By utilizing bootstrapping and topological network decay analysis, we avoided cohort-specific overfitting and identified mathematically stable, biologically rational drivers of progression. However, our analyses rely on retrospective cohorts; prospective clinical validation is necessary to establish the utility of the Rewiring Score. Additionally, while our integrative approach strongly infers a causal trajectory connecting *ZNF268* epigenetic silencing, network rewiring, and metabolic hyperactivation, functional in vivo experiments are required to directly confirm these mechanistic interactions. Another limitation of this study is our reliance on bulk multi-omics data, where variations in tumor purity and cellular composition can act as potential confounders. While we attempted adjustments for these confounders, residual signals from the heterogeneous tissue microenvironment could still influence bulk sequencing matrices. However, because our 10-gene signature captures localized network rewiring rather than broad, non-specific expression shifts, this microenvironmental confounding is likely minimized. Furthermore, our drug-sensitivity analyses are exploratory in nature and require experimental validation. Moreover, cross-platform differences between microarray-based (MSKCC) and RNA-Seq cohorts (TCGA-PRAD and Long et al.) introduce variations in probe dynamic range and background noise, requiring network recalibration for MSKCC. Additionally, external public cohorts are often constrained by missing clinical annotations needed for standard NCCN risk stratification, as well as low patient numbers within risk-stratified groups, which reduces the statistical power of unadjusted Kaplan-Meier log-rank tests. At present, the prostate cancer research community faces a severe data infrastructure constraint: outside of the TCGA-PRAD repository, large-scale public cohorts of localized, primary-site prostate cancer that simultaneously pair modern transcriptomic sequencing (RNA-Seq) with long-term progression-free survival endpoints are virtually non-existent. Consequently, while independent prospective sequencing trials and localized tissue-slice assays represent the ultimate long-term solutions to validate this 10-gene framework, our current multi-cohort validation establishes a robust, mathematically optimized baseline utilizing the highest-quality global data currently available.

In summary, our integrative framework identifies the dynamic, context-dependent network rewiring of *ZNF268* as a fundamental driver of aggressive PCa. Rather than acting as a static biomarker, the transition of the *ZNF268* regulatory module marks a critical evolutionary bottleneck, defining an active molecular bridging state. Translating these systems-level insights into a risk-stratified clinical signature provides a framework to identify clinically low-risk PCa patients already on an aggressive disease trajectory, enabling earlier risk stratification and potentially more precise therapeutic intervention.

## Methods

### Sex as biological variable

This study included clinical biopsies from only male patients with a diagnosis of PCa, given that this disease arises in the prostate gland, which is a male-specific organ. The findings are, therefore, specific to PCa in males.

### Cohorts and data acquisition

TCGA-PRAD multi-omics data were obtained from The Cancer Genome Atlas (TCGA) Prostate Adenocarcinoma (PRAD) project^26^. This dataset comprises RNA-sequencing (RNA-seq), whole-exome sequencing (WES), and DNA methylation profiles derived from fresh-frozen primary tumor specimens obtained via radical prostatectomy. Samples were restricted to primary adenocarcinomas with matched clinical data, excluding metastatic or normal adjacent tissues (Fig. S2a). Furthermore, we utilized the Memorial Sloan Kettering Cancer Center (MSKCC) and Long et al. PCa cohorts with matched transcriptomic profiles and long-term clinical follow-up data, including Biochemical Recurrence (BCR) (Fig. S2b, S2c)^13,27^. Data were accessed from publicly available repositories following their data usage guidelines^13,28^ (SData 9). As this study involved secondary analysis of de-identified data, additional ethical approval was not required. DNase I hypersensitive sites were obtained from the ENCODE project under accession number ENCSR857UZV (RRID:SCR_006793)^15^. Bigwig data derived from normal epithelium of prostate was obtained from the Gene Expression Omnibus database (RRID:SCR_005012) under accession number GSE186458^14^.

### Patient stratification and risk classification

Patients were stratified into risk groups based on NCCN guidelines by three key clinical variables, including Gleason score, preoperative Prostate-Specific Antigen (PSA) levels, and clinical Tumor-stage^4^. High/Very High-risk patients were assigned to this group if they presented with any of the following aggressive characteristics: a Gleason score ≥ 8, a PSA level ≥ 20 ng/mL, or a clinical Tumor-stage of T3 to T4. Low/Intermediate-risk patients were assigned to this group characterized by a Gleason score < 8, PSA levels < 20 ng/mL (stratified as < 10 ng/mL or 10–20 ng/mL), and a clinical Tumor-stage of T1 to T2c (Fig. S1a, Fig. S2a, S2b). In the event of missing information, patients were assigned to the most closely representative groups as defined by the available remaining clinical data. For instance, a patient lacking PSA but with Gleason score 6 and clinical Tumor-stage T2a would be classified as Low/Intermediate-risk. A total of 12 TCGA patients and two MSKCC patients were imputed to the closest NCCN group. For the Long et al. cohort, since there was a lack of clinical information to assign patients according to NCCN guidelines, we categorized the patients into localized and advanced risk groups based on their pathological Tumor-stage (Fig. S2c).

### Differential expression and functional enrichment analysis

Differential gene expression analysis was performed on RNA-seq raw feature counts using the *DESeq2* R package (version 1.52.0, RRID:SCR_015687)^29^. Genes were considered significantly differentially expressed if they exhibited a fold change (FC) ≥ 2 or ≤ −2 with a False Discovery Rate (FDR) ≤ 0.05.

To characterize biological functions associated with differentially expressed genes, we conducted Gene Set Enrichment Analysis (GSEA) and Gene Ontology (GO) analyses using *fgsea* package (version 1.38.0) in R, with default parameters^30^. Hallmark gene sets were obtained from the Molecular Signatures Database (MSigDB) using the msigdbr package (version 26.1.0, RRID:SCR_016863^31^. To preserve both the direction and statistical strength of differential expression genes, unique genes to each risk group were ranked by a significance metric defined as: $\mathrm{Rank}\,\mathrm{Score}=\mathrm{sign}\left({\log }_{2}\left(\mathrm{FC}\right)\right)\times -{\log }_{10}\left(\mathrm{FDR}\right)$ *p*-values were adjusted for multiple testing using the Benjamini-Hochberg procedure, and pathways with an FDR < 0.05 were considered statistically significant.

### Genomic and drug sensitivity analyses

Somatic genomic copy number alterations (CNA) were analyzed using the GISTIC 2.0 algorithm (RRID:SCR_000151), which identifies genomic regions that are significantly amplified or deleted across a set of samples^32^. For this study, GISTIC data were averaged across samples within each risk group to characterize group-specific CNA profiles. The landscape of somatic mutations was further interrogated to identify potential driver genes using Oncodrive analysis to detect non-random mutational clustering. Analysis of somatic variants was conducted using the *maftools* R package (version 2.28.0, RRID:SCR_024519)^33^. Furthermore, clinically actionable alterations were identified by mapping somatic variants to known druggable targets, and operative mutational processes were deconvoluted to characterize specific mutational signatures. Finally, DNA methylation profiles were analyzed using beta values, representing the ratio of methylated probe intensity to the total signal intensity ranging from 0 (unmethylated) to 1 (fully methylated)^34^. Five outlier samples (CNA = 2, DNA Methylation = 3) with measurements exceeding the upper quartile range by approximately ten times have been excluded from the analyses.

### Network proximity analysis between target genes and candidate drugs

To assess the biological plausibility of the candidate drugs within a human interactome framework, we evaluated the network topological proximity between the target cancer gene and the known target proteins of each candidate drug using the method described by Guney et al.^18^. Drug-target interaction profiles for the candidate drugs were retrieved from the Drug-Gene Interaction Database (DGIdb, RRID:SCR_006608)^35^ in addition to target gene annotations from the Genomics of Drug Sensitivity in Cancer (GDSC, RRID:SCR_011956) database^17^. The human protein-protein interaction (PPI) network was retrieved from the STRING database (version 12, RRID:SCR_005223)^36^. To ensure high-confidence interactions, edges were filtered to retain only those with a combined confidence score > 700.

For each drug, T represents the set of drug target proteins and S represents the target disease gene product (S = *ZNF268*). The shortest path network distance *d (s,t)* between drug targets t∈T and s∈S was calculated using the closest distance metric *d*_c_:

2 $${d}_{c}\left(S,T\right)=\frac{1}{|T|}\sum _{t\in T}\mathop{\min }\limits_{s\in S}d\left(s,t\right)$$

To determine whether the observed network distance (${d}_{{obs}}$) was significantly closer than expected by random chance, we generated a degree-preserved null distribution (${d}_{{null}}$) for each drug using 500 random permutations. In each permutation, random target sets matching the node degree distribution of the original drug target set T were selected across the interactome. The proximity Z-Score was calculated as [3]:

3 $$Z=\frac{{d}_{{obs}}-{\mu }_{{null}}}{{\sigma }_{{null}}}$$

where ${\mu }_{{null}}$ and ${\sigma }_{{null}}$ represent the mean and standard deviation of ${d}_{{null}}$ across the 500 permutations, respectively. The *p*-value was calculated as the proportion of permutations where ${d}_{{null}}\le {d}_{{obs}}$. Drugs exhibiting Z ≤ −2.0 and *p* < 0.05 were considered to have statistically significant network proximity to *ZNF268*.

### Weighted graph machine learning model development and feature selection

To prioritize candidate driver genes in PCa, we built separate functional gene networks for high- and low-risk patient groups and then ranked genes based on their structural importance within each network.

The initial discovery and topology-based ranking phase utilize somatic mutation data from the TCGA-PRAD, stratified into a High-Risk network cohort (*N* = 222 samples) and a Low-Risk network cohort (*N* = 276 samples). Raw variants are filtered via strict consequence type matrices to exclude silent mutations, restricted to missense_variant, frameshift_variant, stop_gained, splice_region_variant, inframe_deletion, splice_donor_variant, splice_acceptor_variant, and stop_lost. The sub-networks are subsequently initialized using a seed composed of the top 200 most frequently mutated protein-coding genes. To construct the network boundaries while bypassing database annotation noise, the interactome is expanded via the UniProt-GOA database^37^ and restricted by an intermediate Information Content filtration gateway (1.7 ≤ IC $\le \,$3.7), effectively eliminating uninformative parental terms and hyper-isolated processes. Then we created a High-Risk functional graph containing 12,352 nodes and a Low-Risk graph containing 9654 nodes. From these networks, five centrality-based features are extracted for every gene node, normalized via a standard Z-score scaler, and evaluated using an unsupervised Laplacian Score algorithm to output a structurally ranked candidate gene list based on geometric manifold preservation. To ensure the mathematical validity of this unsupervised ranking discovery step before passing features to downstream modeling, we rigorously validated its performance against the benchmark Cancer Gene Census (CGC) dataset. The framework achieved an Area Under the Receiver Operating Characteristic curve (AUC) of 0.712, demonstrating strong topological classification power. Furthermore, a Precision@K^38^ evaluation against the CGC reference yielded highly accurate target capturing, returning 5, 7, 18, and 38 true positive cancer-associated drivers at K thresholds of 10, 20, 50, and 100, respectively.

All topological network metrics in this pipeline are computed directly on weighted graphs. In these models, the connection strength (edge weight, ${W}_{{ij}}$) between any two genes (nodes) is determined by the number of intermediate-specific Gene Ontology (GO) biological processes they share. For each gene in the High-Risk network (12,352 genes) and the Low-Risk network (9,654 genes), we extract five specific graph connectivity features.

Before applying machine learning, we normalized the centrality metrics so that differences in their value ranges did not dominate the analysis and the metrics could be compared on a similar scale. The raw extracted feature matrix is standardized using a z-score normalization framework via a standard scaler, transforming the features to exhibit a mean of zero and a variance of one (μ= 0, ${\sigma }^{2}\,$= 1). This standardized matrix, where rows represent gene nodes and columns represent the five normalized topological metrics, serves as the exact input matrix for the unsupervised Laplacian Score algorithm. The algorithm evaluates each topological feature’s ability to preserve local graph geometry by constructing a similarity matrix using a nearest-neighbor graph layout. Features and nodes are then ranked by their composite structural importance across these normalized parameters, allowing the machine learning engine to assess the entire network manifold simultaneously and isolate candidate biomarkers that accurately reflect the complex structural landscape of the disease.

To identify robust prognostic markers among the high-risk gene candidates identified by our weighted graph-based machine learning model, we first selected genes showing significantly different expression between patients in the Low/Intermediate-risk and High/Very High-risk groups. We then employed a stability selection approach using Least Absolute Shrinkage Selection Operator (LASSO) Cox regression (*glmnet* R package, version 5.0, RRID:SCR_015505)^39,40^ with an alpha parameter of 1. A 10-fold cross-validation was performed to determine the optimal tuning parameter (λMin), and features with non-zero coefficients were extracted. To ensure the stability and reproducibility of the selected features, we performed a bootstrap-based stability selection procedure with 500 iterations. The gene expression data were first standardized (z-score normalization) to ensure comparable scales across all predictors. Genes were considered “stable” if they were selected in more than 80% of the bootstrap iterations (Selection Frequency > 0.8). Figure S14 provides an overview of the study, including our machine learning approach.

### Co-expression network construction

To reduce low-variance background noise, RNA-seq matrices were filtered to retain the top 5000 most highly variable genes across the cohort along with the core gene, *ZNF268*. For baseline co-expression visualization, expression data were evaluated utilizing the *corrr* R package (version 0.4.5) to extract the top genes most highly correlated with *ZNF268* across risk subgroups. Network topological stringency and module rigidity were assessed by plotting the decay of absolute Pearson correlation coefficients across genes ranked by their correlation value to identify connectivity thresholds.

### Topological network rewiring

We applied a Weighted Gene Co-expression Network Analysis (*WGCNA*, R package version 1.74, RRID:SCR_003302) framework^41^ to quantitatively map structural network alterations. Log2-transformed expression data were converted into adjacency matrices utilizing a soft-thresholding power of 6 based on the standard network topology criterion proposed by Langfelder and Horvath^41^, which were subsequently transformed into Topological Overlap Matrices (TOM). Scale-free topology analysis demonstrated that a power of 6 represented the lowest threshold where the scale-free topology fit index (R^2^) successfully cleared the standard benchmark of 0.80 while simultaneously maintaining a high mean network connectivity. This optimization kept the equilibrium between biological signal capturing and scale-free network architecture topology.

We calculated the Network rewiring (Δ connectivity) for *ZNF268* by isolating its TOM similarity vector in both the High *ZNF268* and Low *ZNF268* states of the Low/Intermediate-risk group and computing the absolute difference between the two states.

### Rewiring Score development

We divided the TCGA-PRAD into two independent 50/50 data partitions, and we performed the TOM analyses for 100 iterations per split to prevent overfitting. Furthermore, we selected the genes identified in this process and that stayed stable more than 80% through the iterations and shared between two splits as the core genes to develop “Rewiring Score” [4].

The Rewiring Score is formulated as a prognostic index, where the expression levels (*E*) of each gene in our signature are multiplied by their respective Cox regression coefficients (β). These coefficients were derived from a multivariable Cox proportional hazards model trained in the TCGA-PRAD cohort and represent the log-hazard ratio for each gene’s contribution to progression. This allows the signature to function as a continuous risk metric, which we subsequently dichotomized into ‘High’ and ‘Low’ scores using an optimal cutpoint approach derived from the PFS survival data in the Low/Intermediate-risk cohort.

4 $${RewiringScor}{e}_{i}=\mathop{\sum }\limits_{j=1}^{N}\left({\beta }_{j}\cdot {E}_{{ij}}\right)$$

The coefficients from TCGA-PRAD were applied directly to the Long et al. cohort, as both gene-expression datasets were generated using RNA sequencing. Because the genes in the MSKCC cohort were profiled using microarrays, we recalibrated the model coefficients to account for cross-platform differences. This recalibration was necessary to mitigate the effects of dynamic range compression and hybridization background noise inherent to legacy microarray platforms, which can disproportionately affect the measurement of low-abundance transcripts^42,43^.

### Statistical analysis

All statistical analyses were conducted using the R statistical software version 4.6.0 (RRID:SCR_001905). Survival analysis was performed using the *survival* package version 3.8-9 in R^44^, and survival probability was visualized using the Kaplan-Meier method. To evaluate our model across the broader timeline of disease progression, we looked at distinct survival endpoints in each cohort. For TCGA-PRAD, the endpoint was Progression-Free Survival (PFS), representing definitive clinical progression events. For the Long et al. and MSKCC cohort, we utilized Biochemical Recurrence (BCR), defined as a postoperative PSA ≥ 0.2 ng/mL or the initiation of salvage therapy. Given that BCR provides an earlier, highly sensitive readout of initial progression compared to the later clinical endpoints captured by PFS, using both metrics to study the robustness of the Rewiring Score across the entire evolutionary spectrum of localized PCa failure. Multivariable analyses were conducted by adjusting for relevant clinical variables available for each cohort. To evaluate context-dependent prognostic utility, formal interaction models were constructed by incorporating an interaction term into the multivariable Cox regression models [5]:

5 $${ZNF}268\,{expression}\,\times \,{NCCN}\,{Risk}\,+\,{Avaialble}\,{Clinical}\,{variables}$$

Following the identification of significant interactions, stratified Kaplan-Meier and Cox proportional hazards analyses were performed independently within the Low/Intermediate and High/Very High clinical risk strata. Detailed results of the survival analyses, including regression coefficients, hazard ratios (HRs), 95% confidence intervals (CIs), standard errors, Wald z-statistics, and *p*-values, are provided in SData 10.

All correlation analyses were performed using Pearson’s method unless otherwise specified. All tests were 2-sided when applicable, and *p* < 0.05 was considered statistically significant. Results were corrected for multiple testing using the Benjamini-Hochberg method (FDR) unless otherwise stated. All measurements were taken from distinct individual samples. Violin and box plots should be interpreted as follows: horizontal lines denote median values; boxes extend from the 25th to the 75th percentile of each group’s distribution of values; vertical extending lines denote adjacent values (the most extreme values within 1.5 interquartile range of the 25th and 75th percentile of each group). Differences between groups were assessed by the Wilcoxon rank test. Significance is indicated as follows in the figures: *p ≤ 0.05; **p ≤ 0.01; ***p ≤ 0.001; ****p ≤ 0.0001.

## Supplementary information

Supplementary information

Supplementary data

## Data Availability

Data for the TCGA-PRAD were accessed from the original publication and available repositories^26,45,46^. The data for the Long et al. and MSKCC cohort were obtained from the original publication and publicly available repositories^13,27,45^. DNase I hypersensitive sites were obtained from the ENCODE (RRID:SCR_006793) project under accession number ENCSR857UZV^15^. BigWig data derived from normal epithelium of prostate was obtained from the Gene Expression Omnibus database under accession number GSE186458^14^.

## Code availability

The code used to generate the results presented in this study is publicly available in the A. Lundberg Lab GitHub repository: https://github.com/arianlundberg/ML_PrimaryPCa.
