# Supplementary material for: A novel multiomics machine learning signature identifies rapid progression in clinically low risk prostate cancer
Source: NPJ Digit Med. 2026 Sep 14;9:702. doi: 10.1038/s41746-026-03254-5 (PMC13575102; doi:10.1038/s41746-026-03254-5)
Supplement: Supplementary file 1 [file 41746_2026_3254_MOESM1_ESM.pdf]

Figure S1

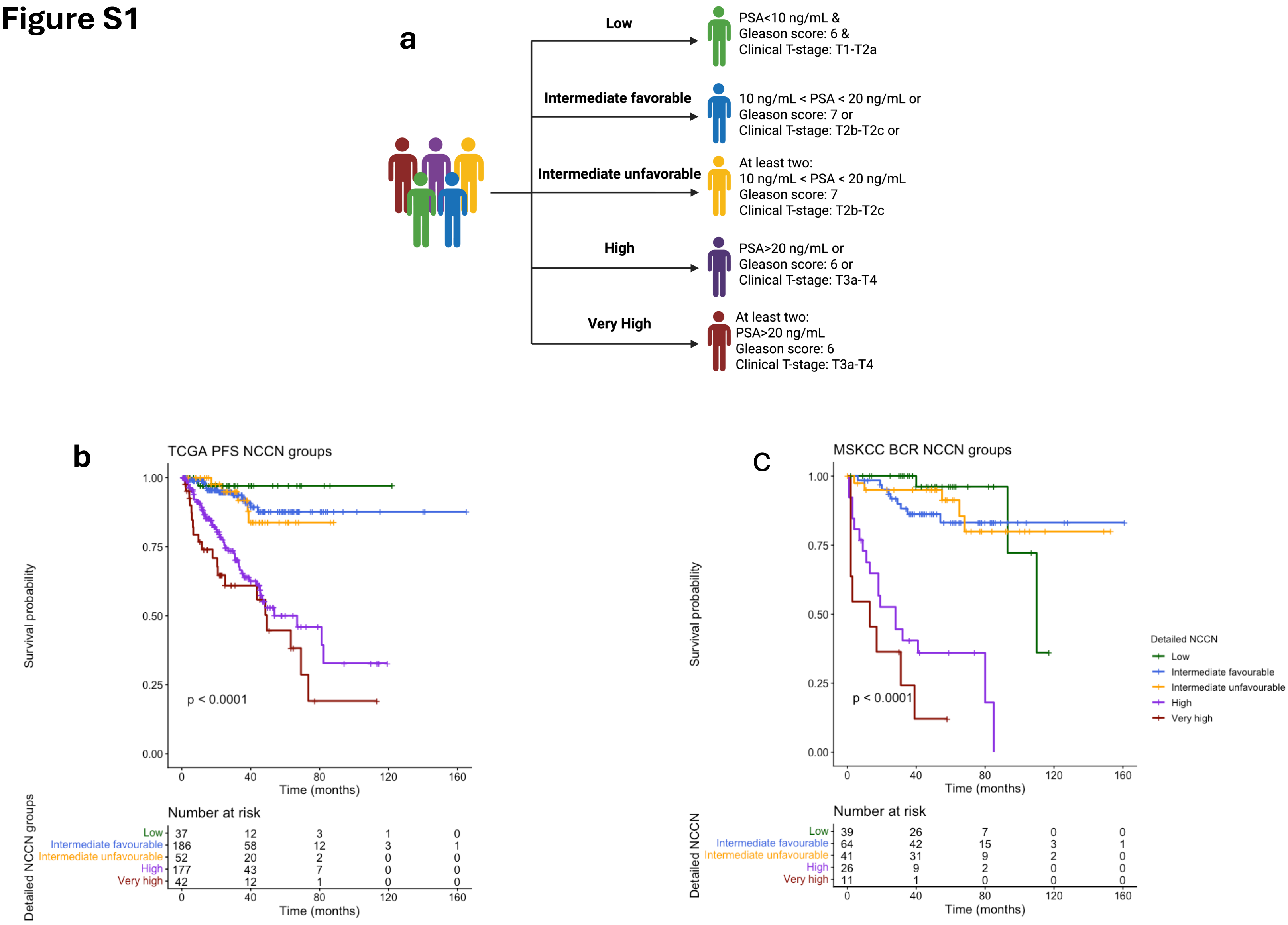

d

Pairwise comparisons using Log-Rank test within NCCN group

TCGA-PRAD

NCCN-risk groups	Low	Intermediate favorable	Intermediate unfavorable	High
Intermediate favorable	0.38123	-	-	-
Intermediate unfavorable	0.36687	0.75589	-	-
High	0.00109	7.8e-09	0.00137	-
Very high	0.00010	9.5e-11	0.00025	0.14293

MSKCC

NCCN-risk groups	Low	Intermediate favorable	Intermediate unfavorable	High
Intermediate favorable	0.45	-	-	-
Intermediate unfavorable	0.87	0.78	-	-
High	4.7e-09	4.7e-09	2.6e-07	-
Very high	1.2e-10	3.8e-10	2.4e-09	0.20

Figure S1. Kaplan-Meier survival analysis for Progression-free survival (PFS) and Biochemical-recurrence (BCR) based on standard NCCN risk guidelines (Low, Intermediate-favorable, Intermediate-unfavorable, High, Very High).
(a) schematic view of NCCN classification; (b-c) Kaplan-Meier survival analysis for PFS and BCR including all risk groups. (d) pairwise comparisons using Log-rank test within NCCN groups.

Figure S2

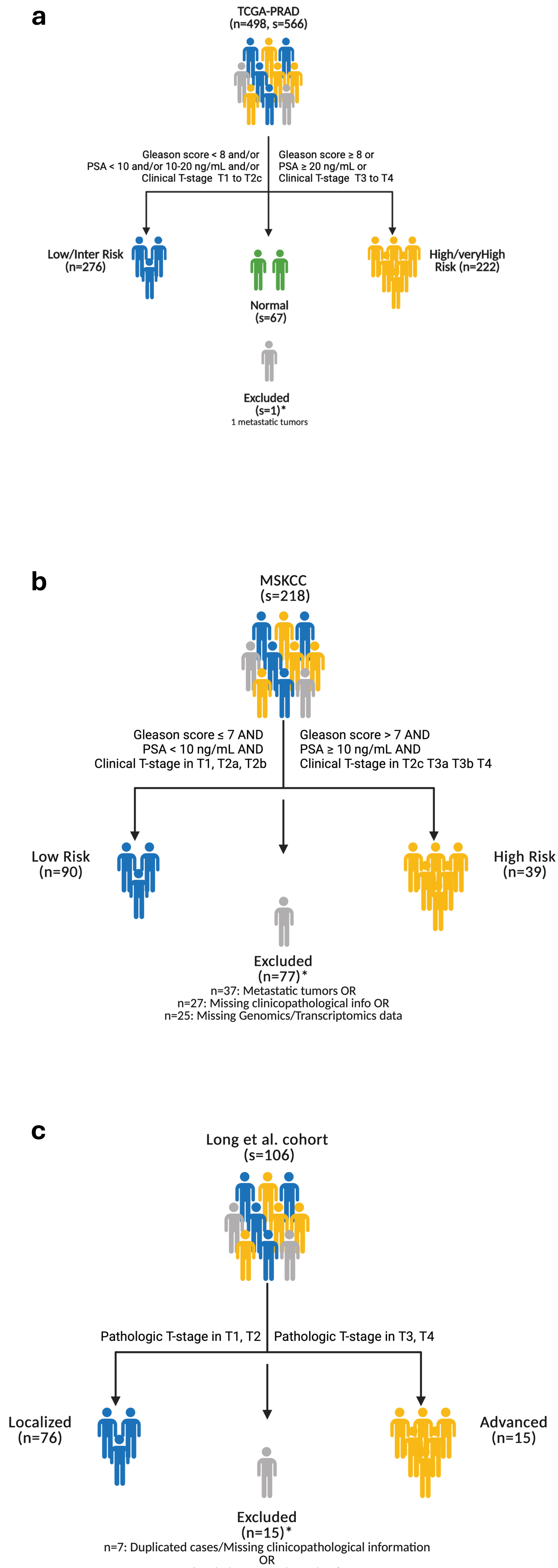

Figure S2. Distribution of the merged clinical strata (Low/Intermediate-risk and High/Very High-risk) in the TCGA-PRAD (a), MSKCC (b) and Long et al. cohorts (c).

Figure S3

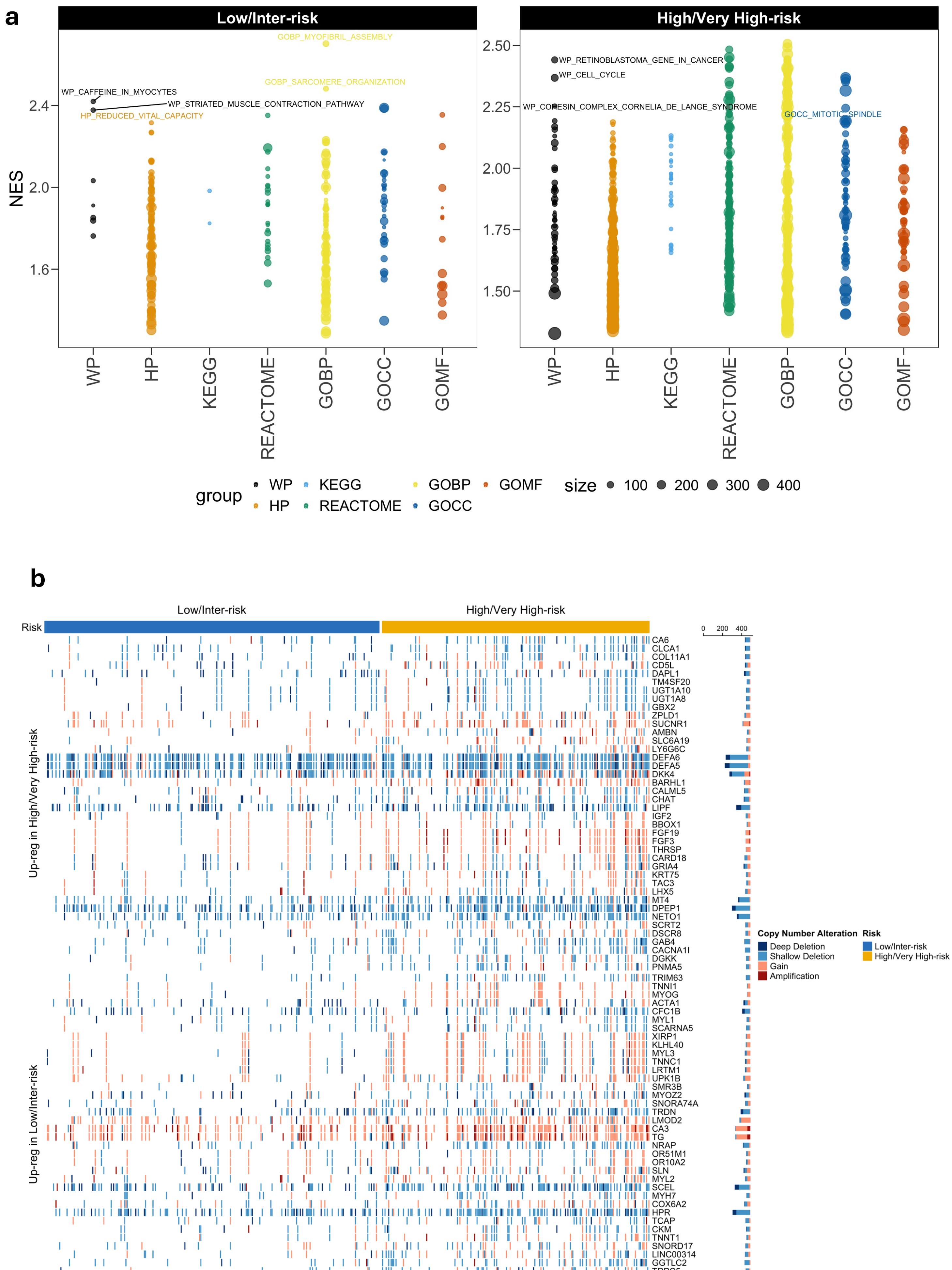

Figure S3. Functional enrichment analysis across GO, KEGG, Reactome, and WikiPathways databases, comparing High/Very High-risk and Low/Intermediate-risk groups.
(a). Heatmap representing somatic copy number status for the top differentially expressed genes across individual patient samples (b).

Figure S4

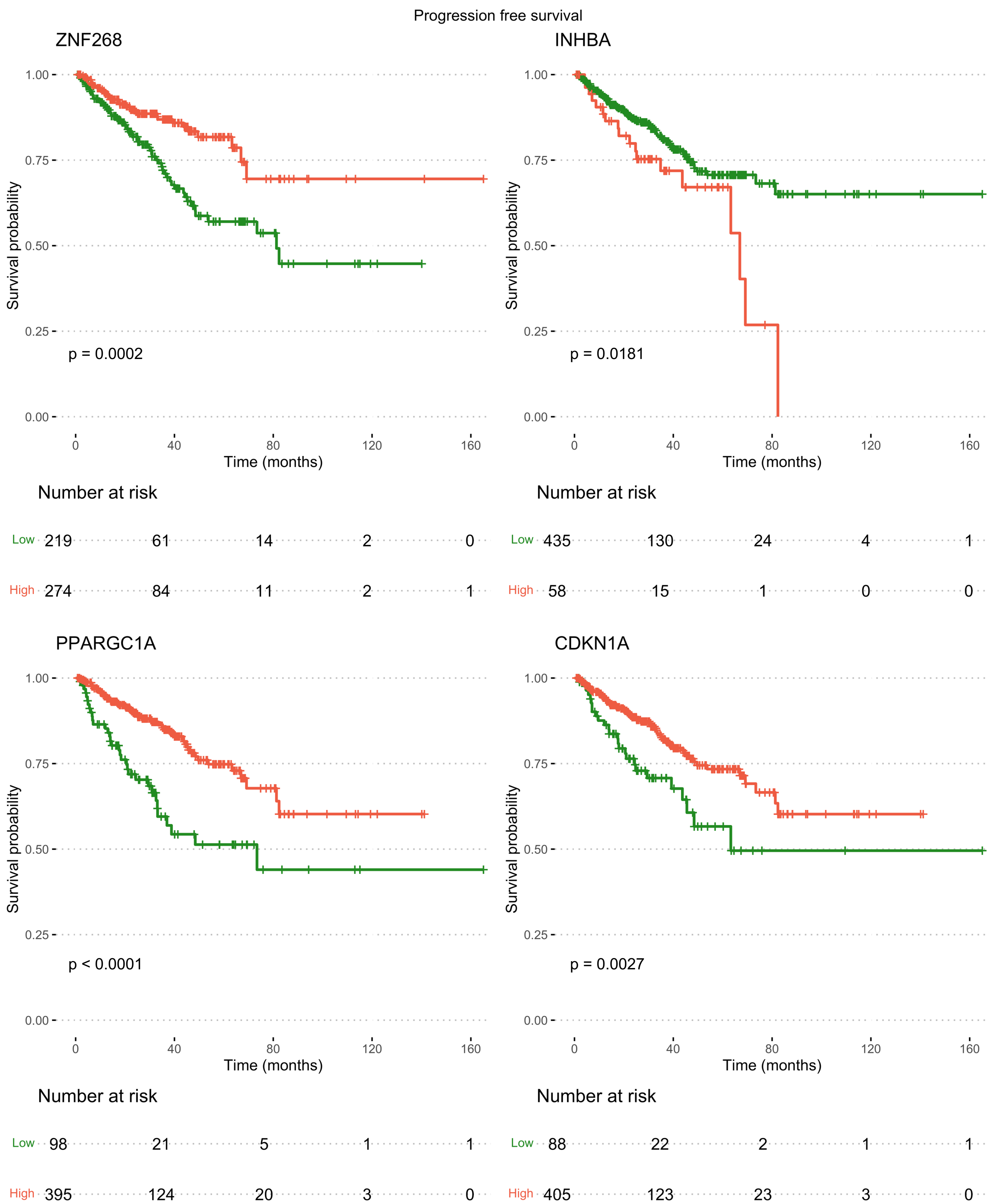

Figure S4. Univariable Kaplan-Meier survival analysis for the individual four critical genes (*ZNF268, PPARGC1A, INHBA, CDKN1A*) isolated via the LASSO Cox regression model.

Figure S5

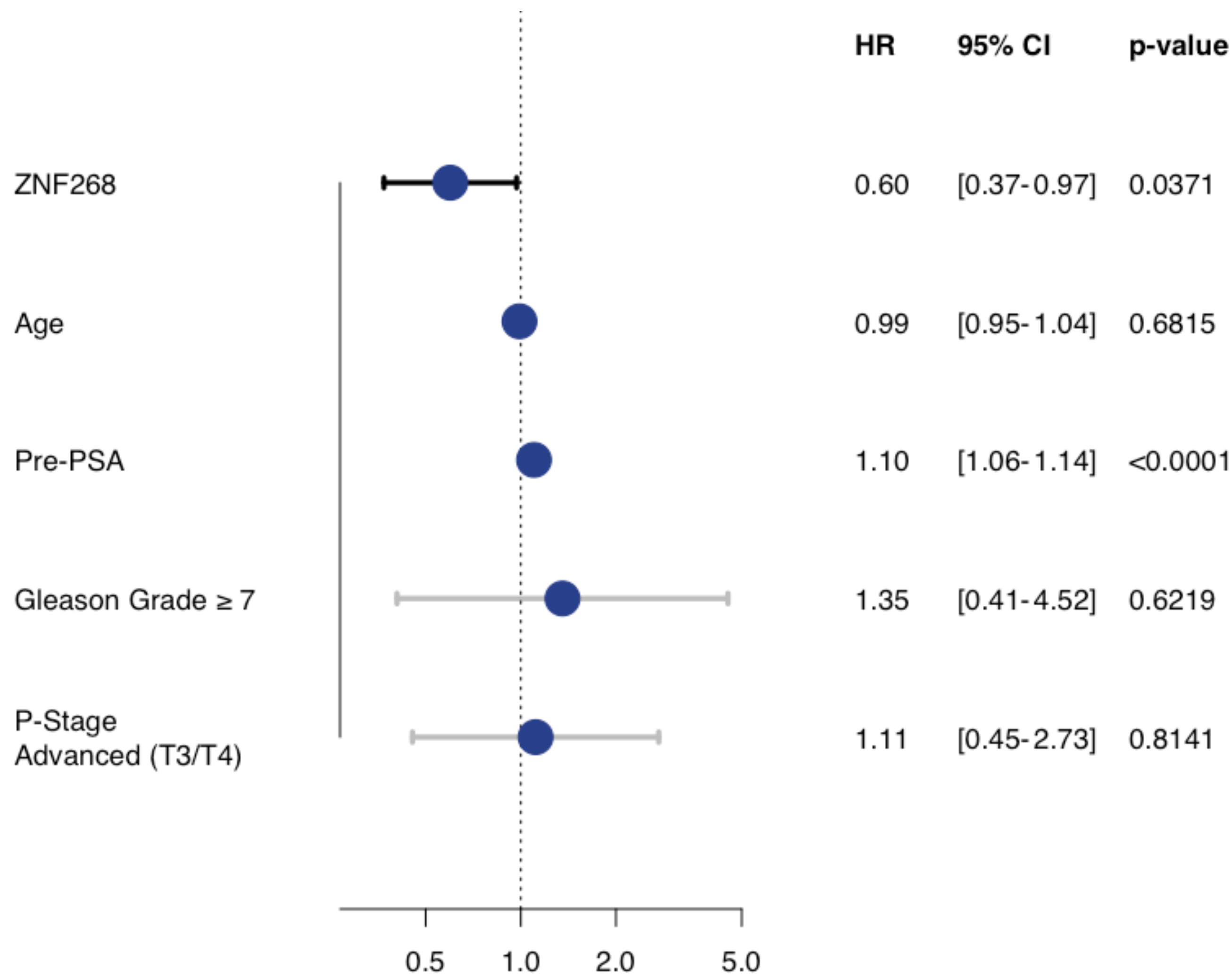

Figure S5. Independent validation of *ZNF268* as a protective prognostic factor in primary prostate cancer.
Forest plot displaying multivariable Cox proportional hazards regression analysis of ZNF268 gene expression alongside baseline clinical and pathological covariates in Long et al. cohort (Cancer Research 2014).

Figure S6

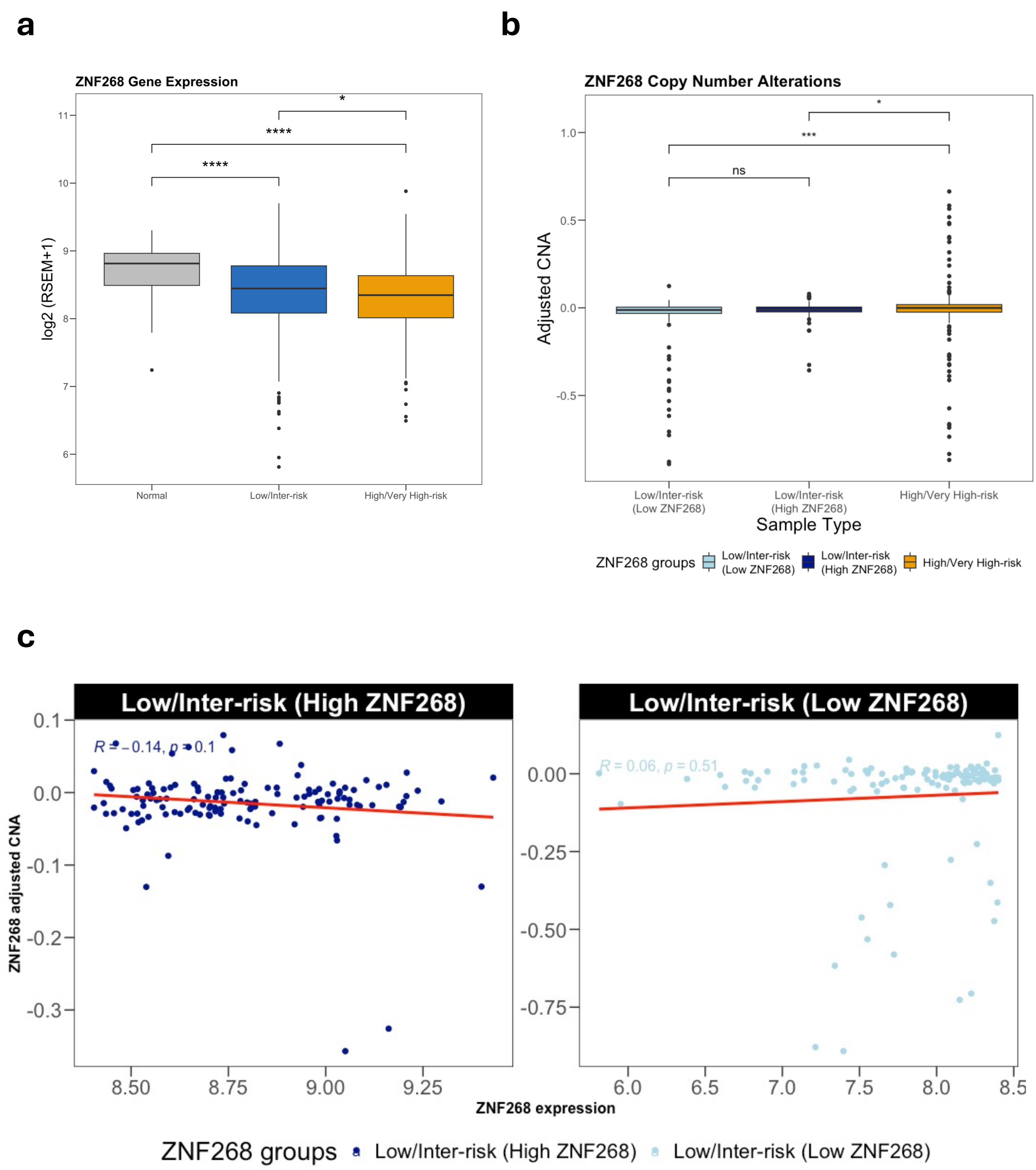

Figure S6. Comprehensive multi-omics evaluation of the *ZNF268* locus.
(a) Stepwise reduction of ZNF268 mRNA expression across clinical disease states (normal prostate tissue to High/Very High-risk tumors). (b) Somatic copy number alterations (CNA) across the defined clinical subgroups. (c) scatterplots representing the correlation between cop between ZNF268 CNA and gene expression.

Figure S7

a

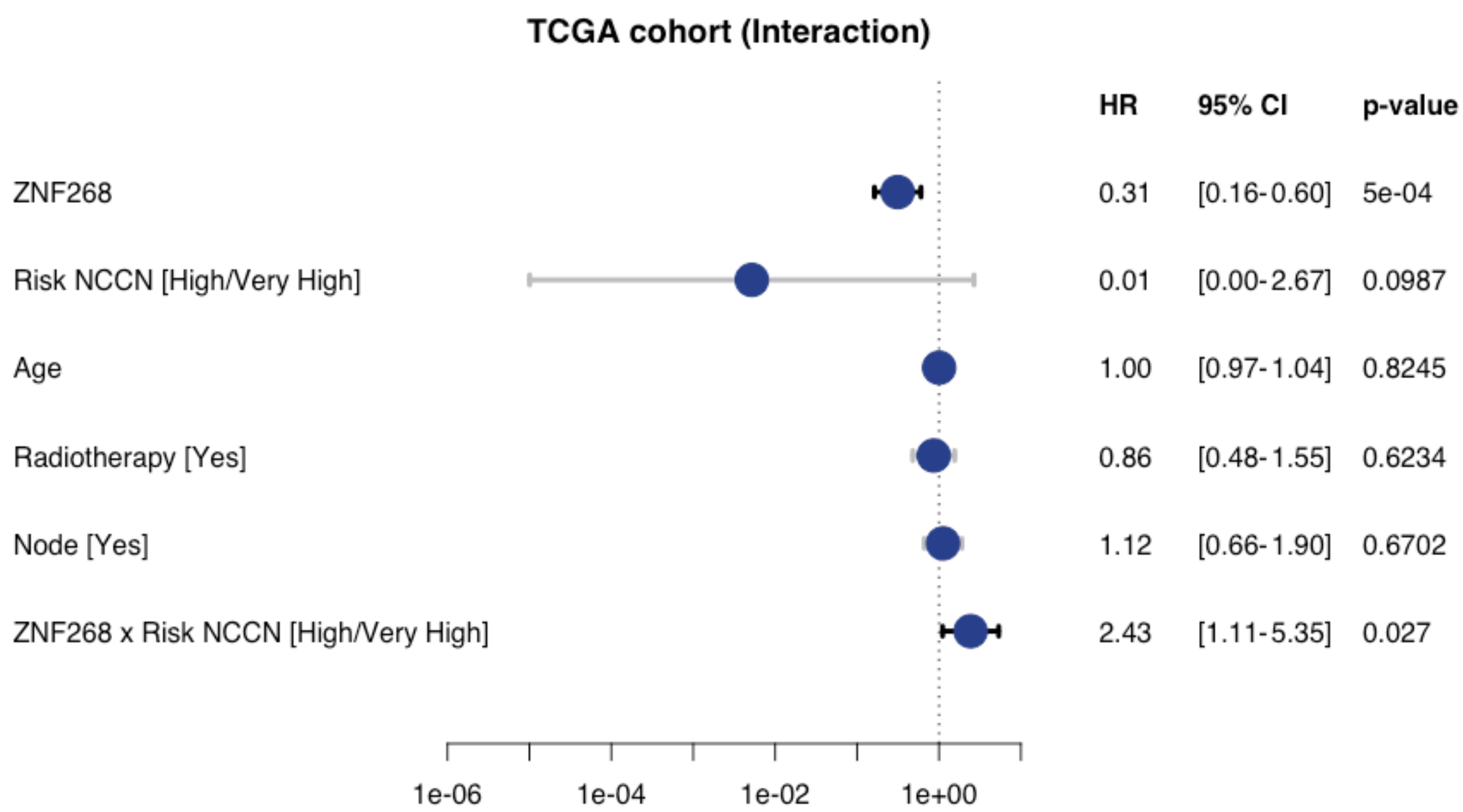

b

Characteristic	HR	95% CI	p-value
ZNF268	0.31	0.16, 0.60	<0.001
NCCN			
Low/Inter	—	—	
High/veryHigh	0.01	0.00, 2.67	0.10
Age	1.00	0.97, 1.04	0.8
Radiotherapy			
No	—	—	
Yes	0.86	0.48, 1.55	0.6
Node			
No	—	—	
Yes	1.12	0.66, 1.90	0.7
ZNF268 * NCCN			
ZNF268 * High/veryHigh	2.43	1.11, 5.35	0.027

Abbreviations: CI = Confidence Interval, HR = Hazard Ratio

Figure S7. Statistical validation of the targeted interaction model (*ZNF268* × NCCN Risk + Clinical variables).
Forestplot (a) and clinicopathological characteristics (b) table representing interaction model between ZNF268 and NCCN risk factors adjusted for available clinical variables available in the TCGA-PRAD cohort.

Figure S8

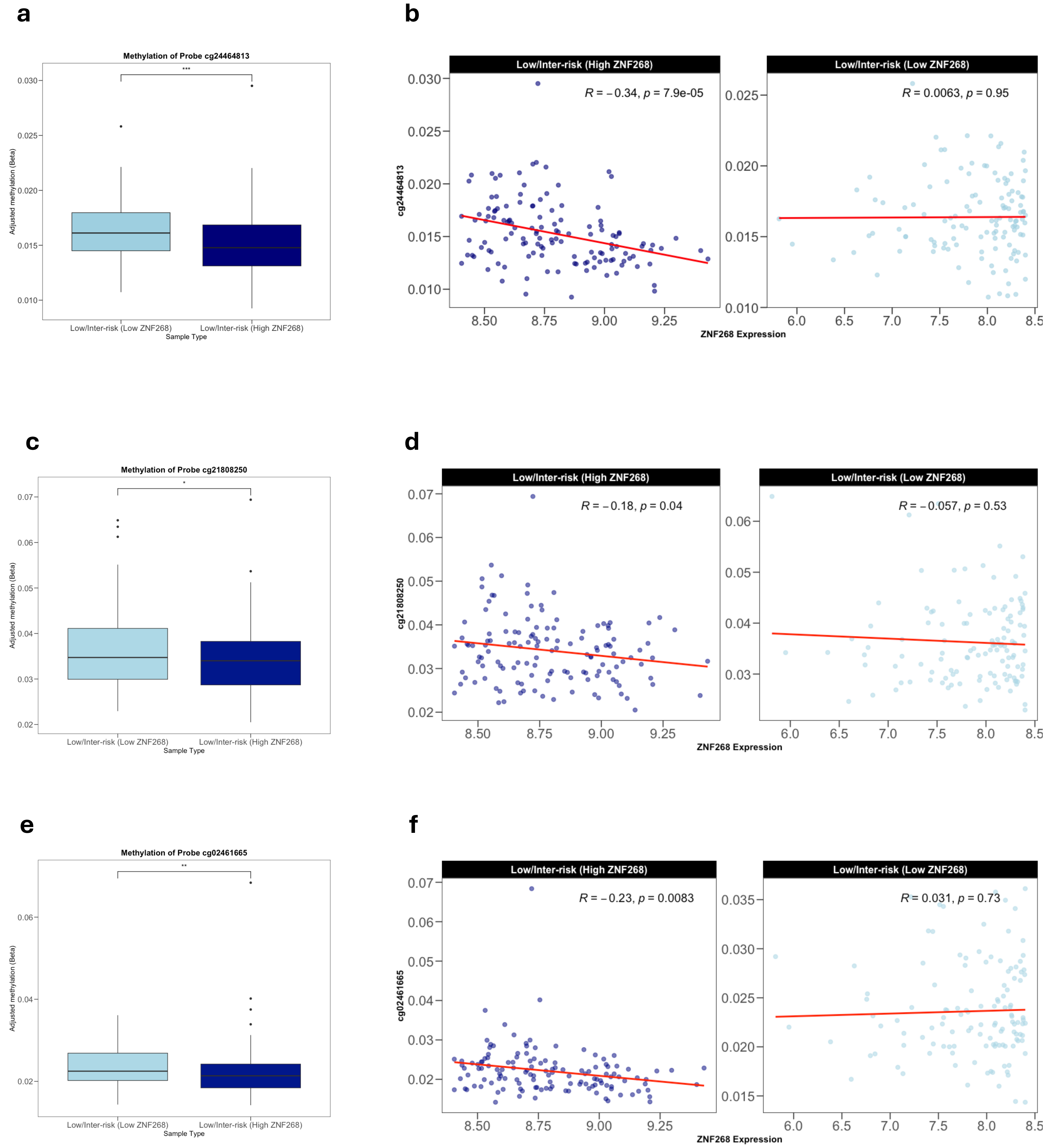

Figure S8. Expanded locus-specific methylation profiling mapping individual CpG probes to *ZNF268* transcript levels.

Boxplots (a, c, e) comparing the adjusted methylation levels within the CpG probes between Low/Inter-risk (Low ZNF268, light blue) and Low/Inter-risk (High ZNF268, dark blue) tumors. Scatterplots (b, d, f) representing the correlation of each CpG probe with ZNF268 gene expression, separated into Low/Inter-risk (Low ZNF268, light blue) and Low/Inter-risk (High ZNF268, dark blue).

Figure S9

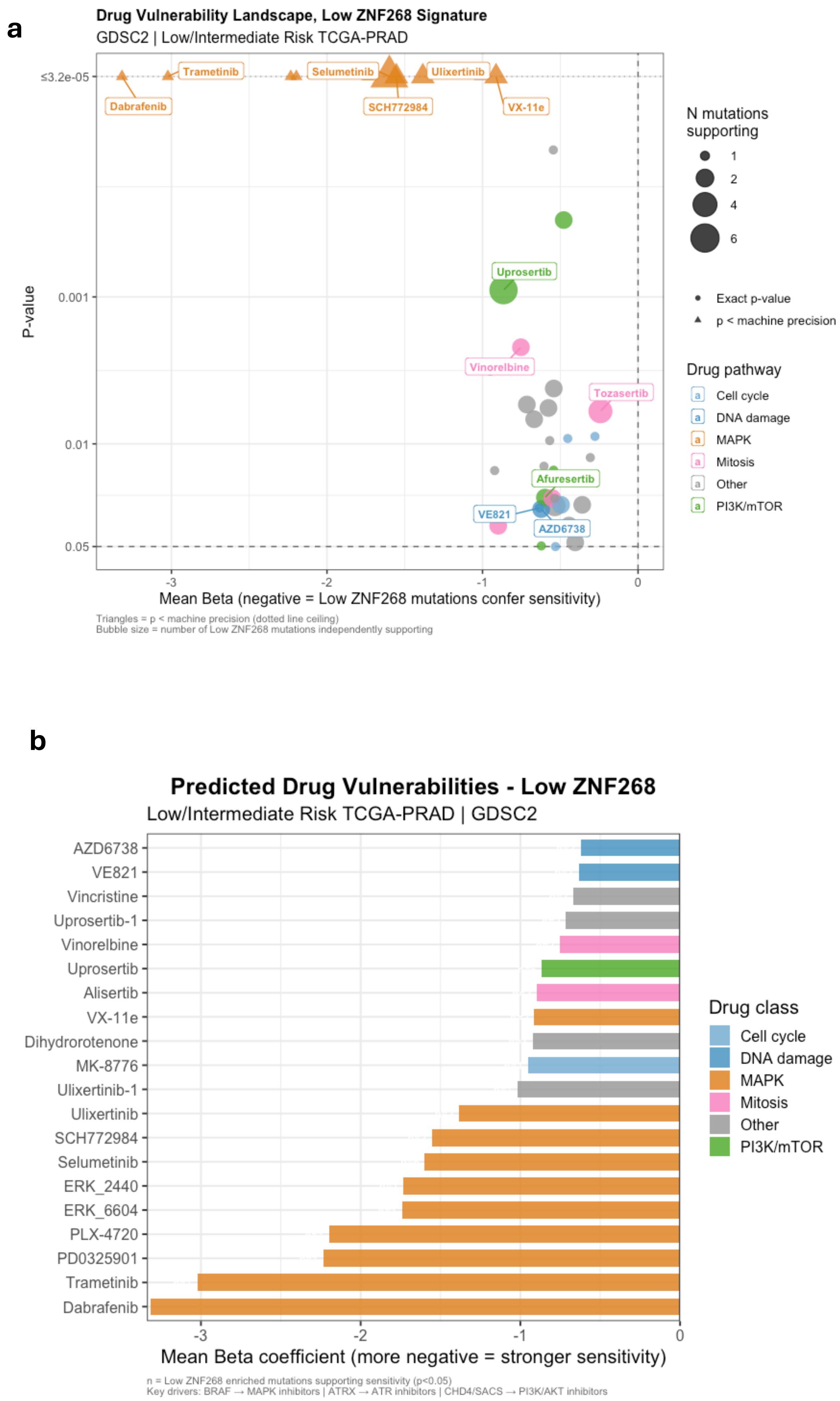

Figure S9. Drug vulnerability landscape in Low ZNF268 group within Low/Inter-risk NCCN patients of TCGA-PRAD.

Figure S10

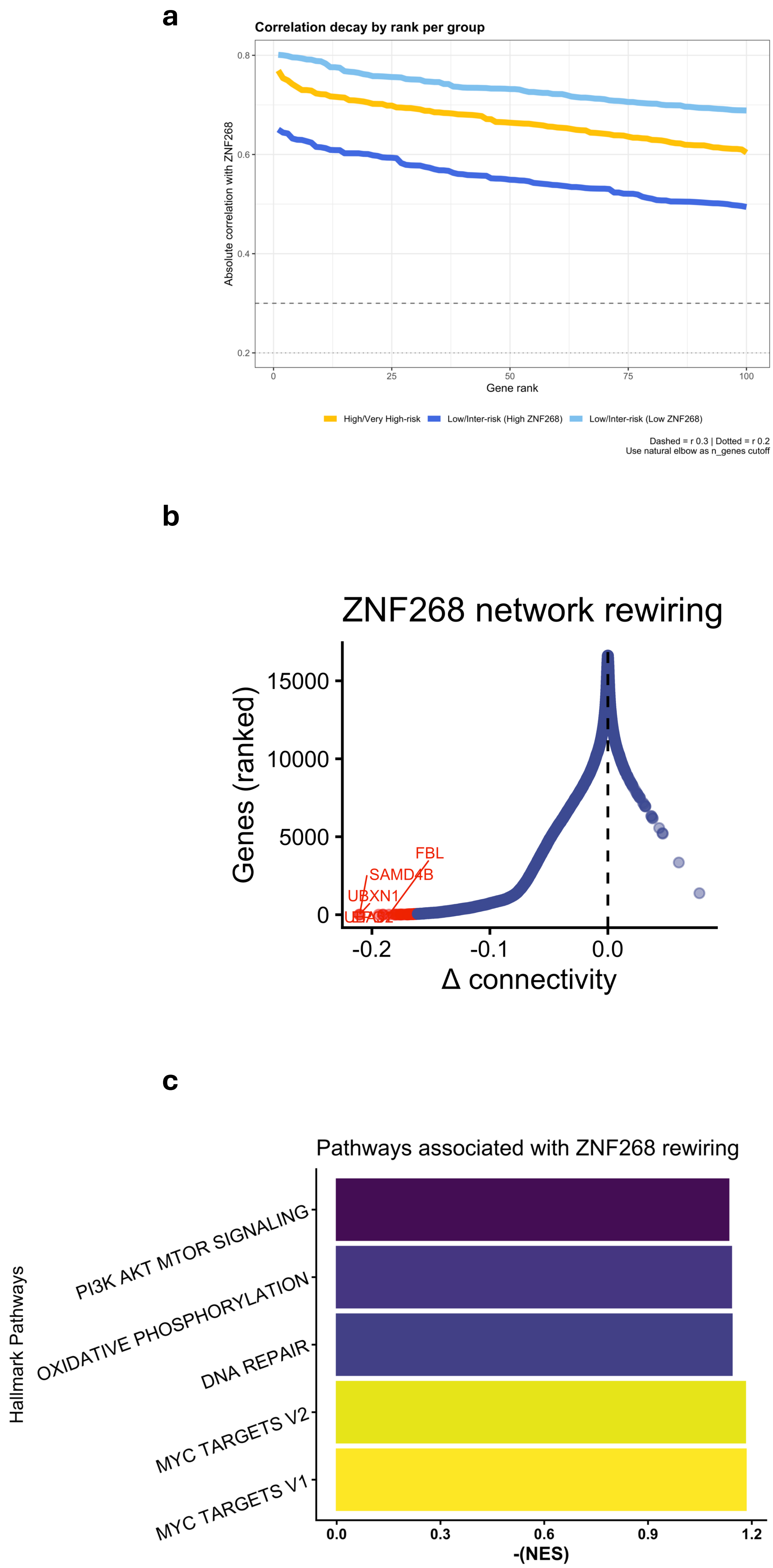

Figure S10. Network correlation decay and rewiring genes connectivity across NCCN groups. a) The slower decay rate in the Low ZNF268 transitional group indicates a rigid, tightly coordinated module characteristic of a molecular bottleneck. b) Network rewiring quantified via differential connectivity (connectivity) between High and Low ZNF268 states within the Low/Intermediate-risk group. (c) Pathway enrichment of highly rewired genes, highlighting functional uncoupling in cancer hallmarks.

Figure S11

a

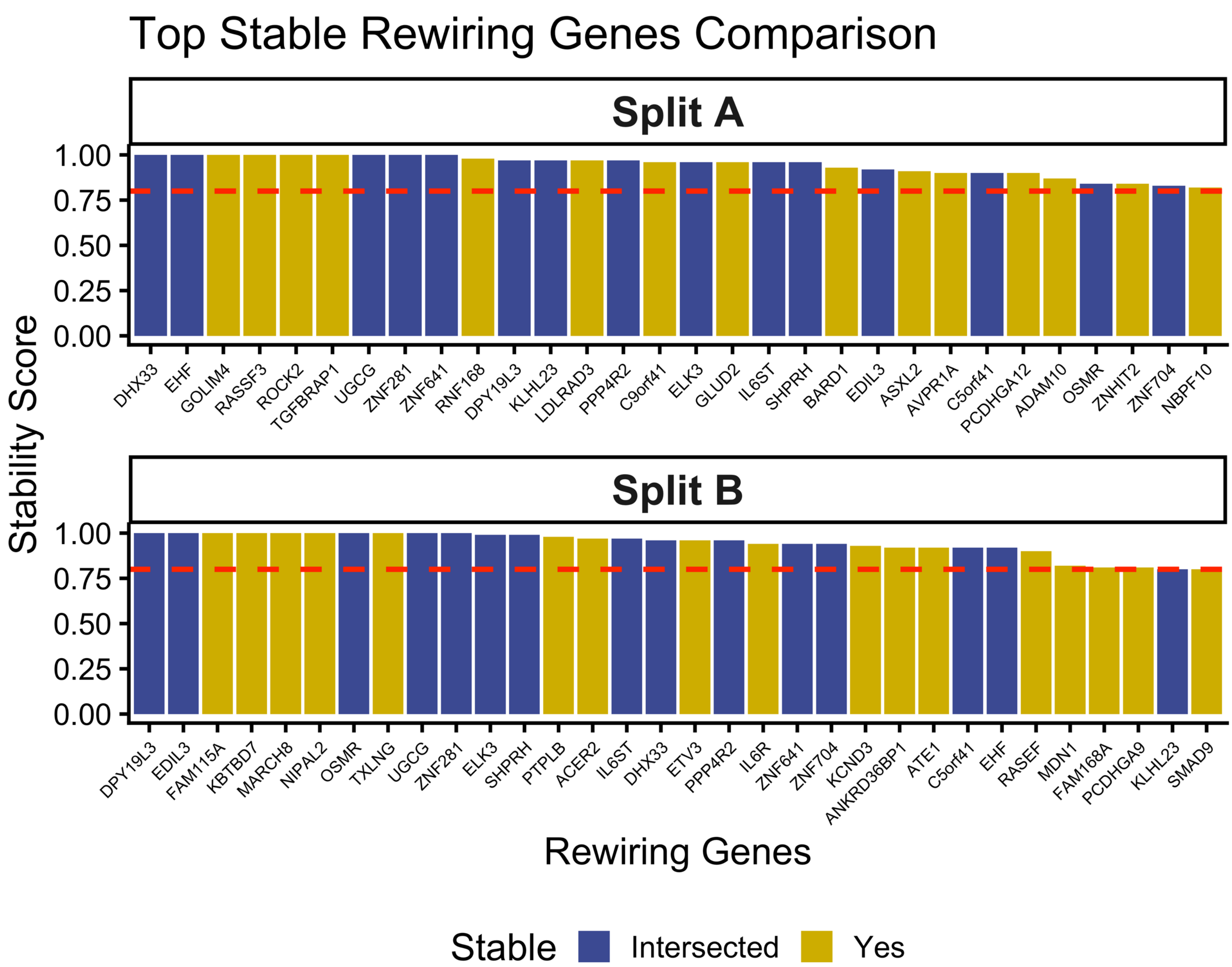

b

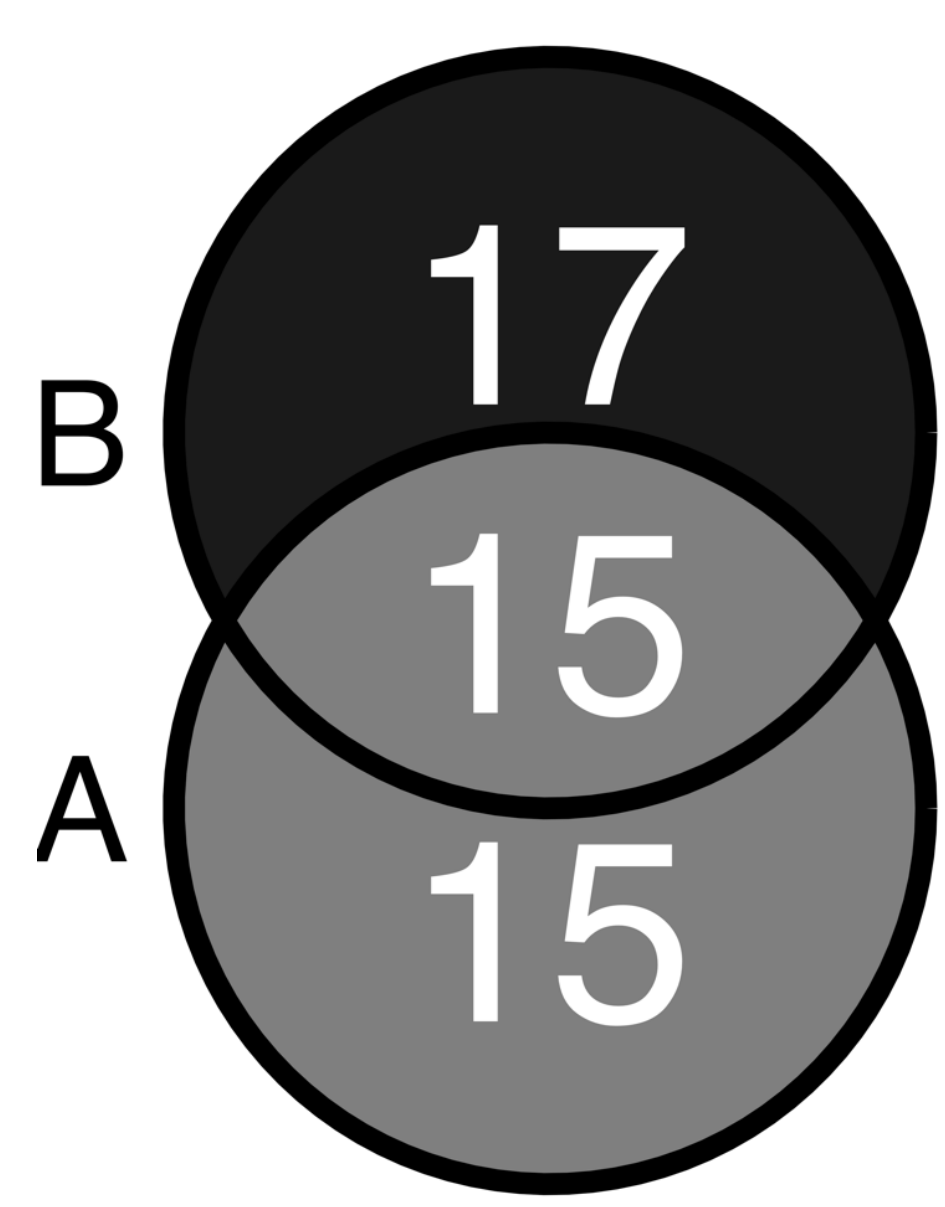

Figure S11. Rewiring score gene selection through stability and posthoc machine learning approach.

a) Barplots representing top stable genes across the two splits of the TCGA-PRAD. Genes passing an 80% stability score in both splits are selected as the rewiring gene candidates (marked in blue). b) Venn diagram representing shared stable genes via parallelized bootstrapping across independent data partitions.

Figure S12

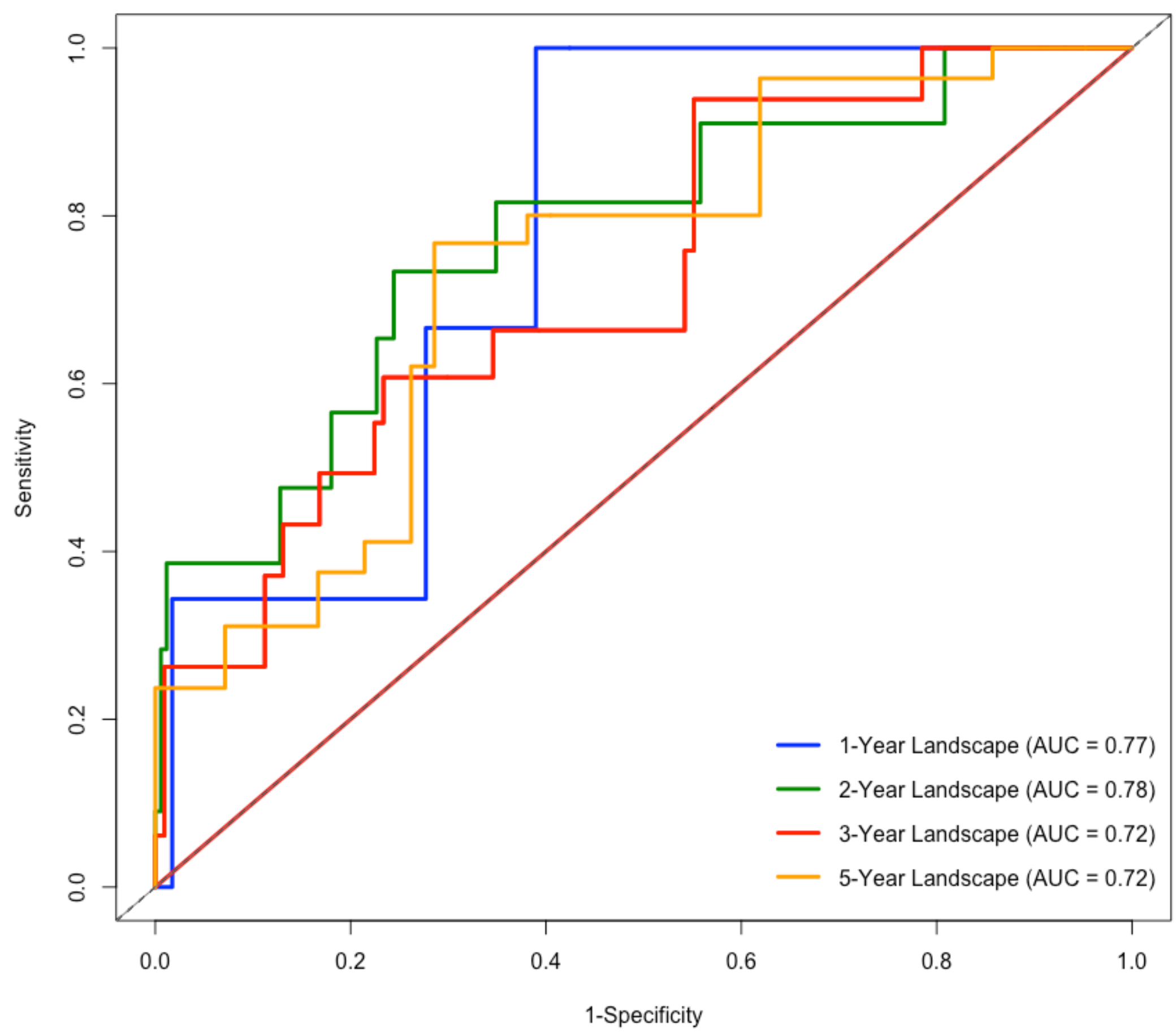

Figure S12. Evaluation of the performance for the rewiring score over time by the time-dependent Area under the curve (AUC) within the low/intermediate risk population.

Figure S13

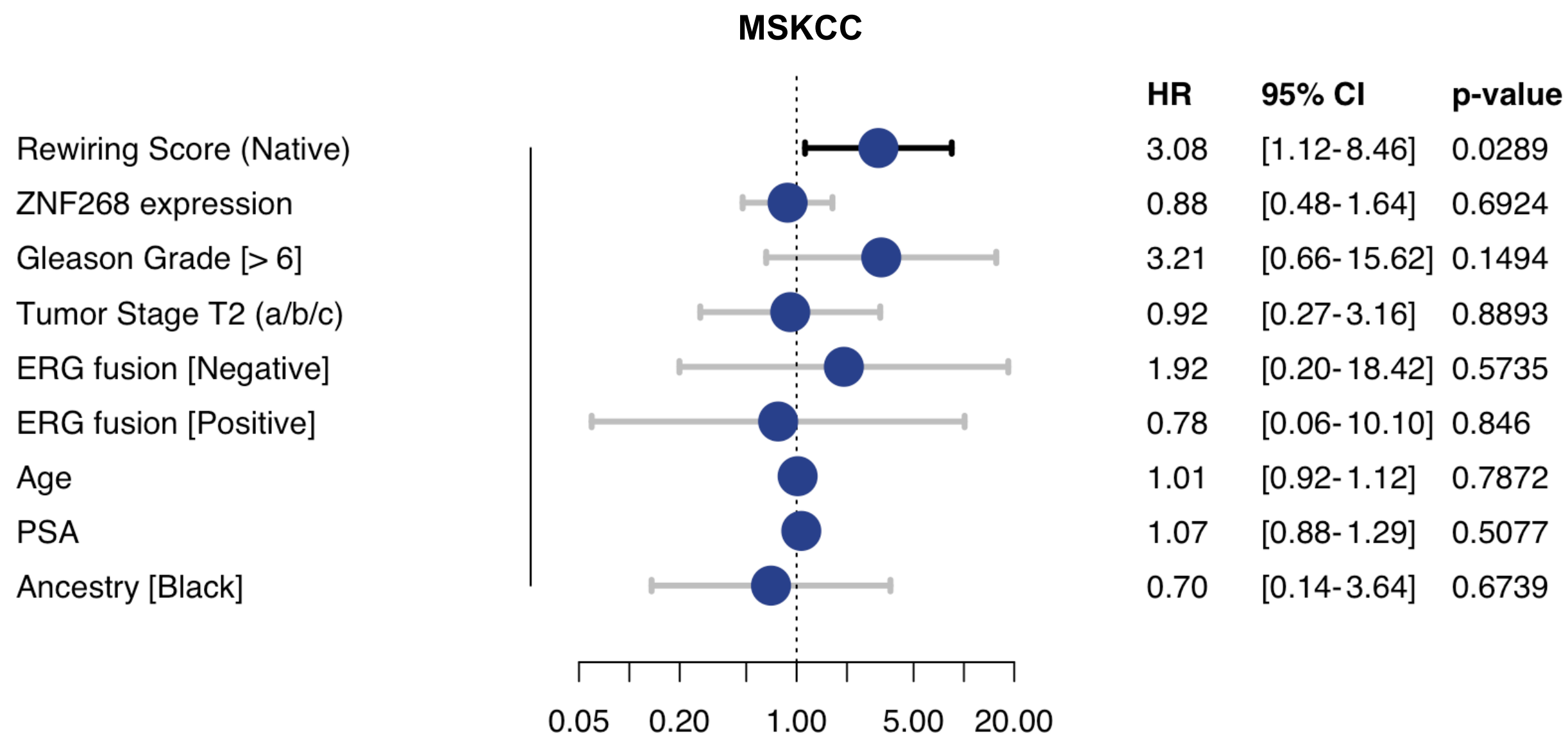

Figure S13. The MSKCC cohort, demonstrating the Rewiring Score predicts Biochemical-recurrence in early-stage disease when adjusted for clinical and molecular covariates.

Figure S14

a

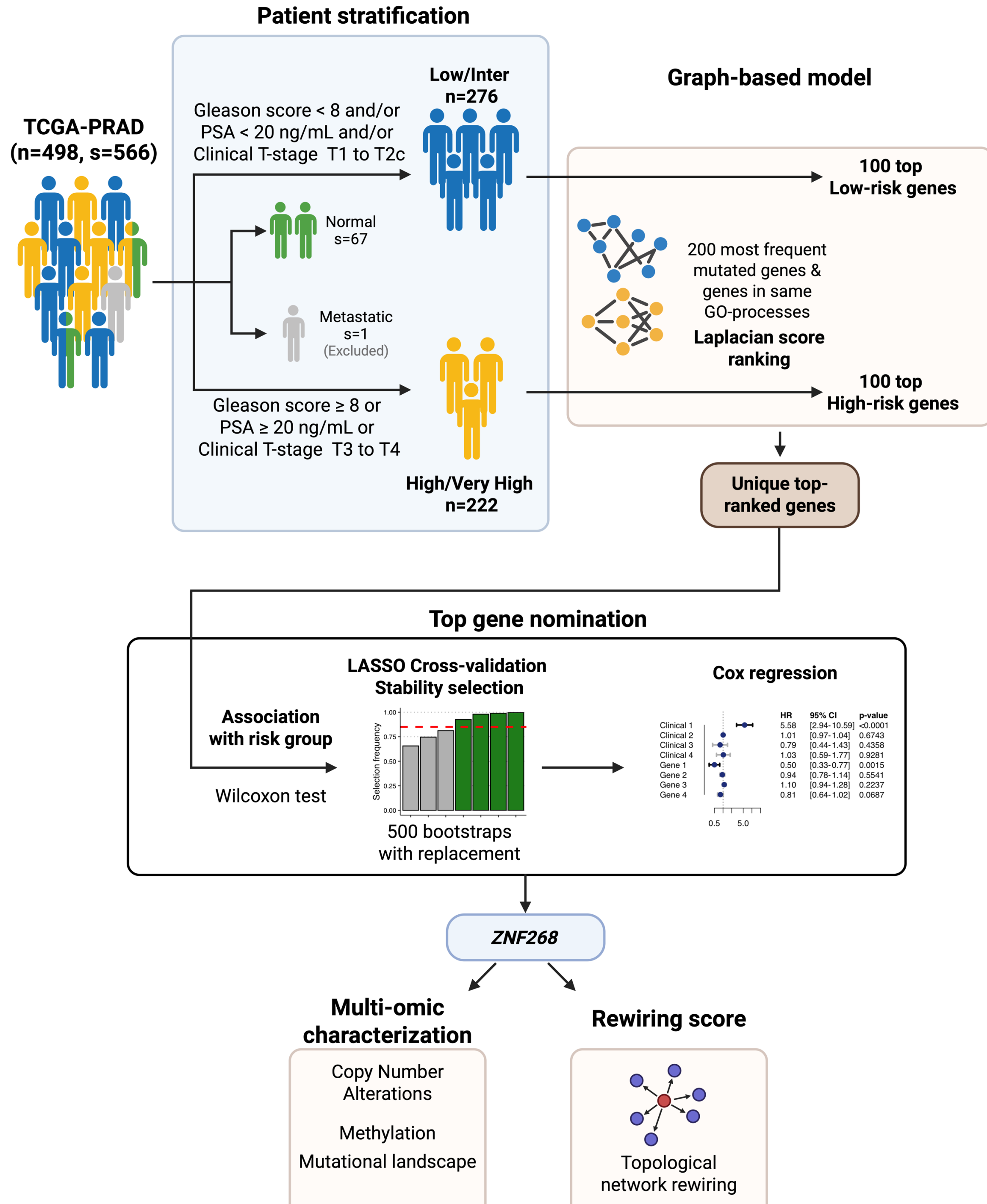

b

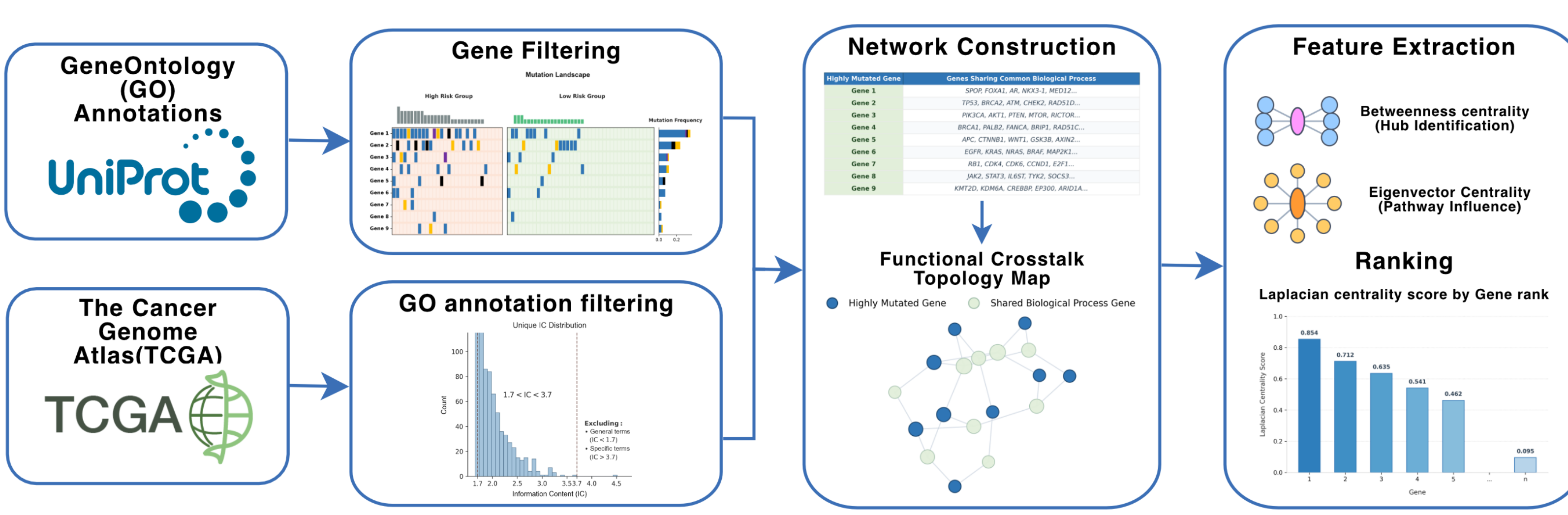

Figure S14. Schematic view of the study framework (a) and the machine learning approach (b)
